# Cytoskeletal organization in isolated plant cells under geometry control

**DOI:** 10.1073/pnas.2003184117

**Published:** 2020-07-08

**Authors:** Pauline Durand-Smet, Tamsin A. Spelman, Elliot M. Meyerowitz, Henrik Jönsson

**Affiliations:** ^a^The Sainsbury Laboratory, University of Cambridge, Cambridge CB2 1LR, United Kingdom;; ^b^Division of Biology and Biological Engineering, California Institute of Technology, Pasadena, CA 91125;; ^c^Howard Hughes Medical Institute, California Institute of Technology, Pasadena, CA 91125;; ^d^Department of Applied Mathematics and Theoretical Physics, University of Cambridge, Cambridge CB3 0WA, United Kingdom;; ^e^Department of Astronomy and Theoretical Physics, Computational Biology and Biological Physics, Lund University, 221 00 Lund, Sweden

**Keywords:** cytoskeleton, microtubules, plant cells, actin, cell geometry

## Abstract

The cytoskeleton plays a key role in establishing robust cell shape. In animals, it is well established that cell shape can also influence cytoskeletal organization. Cytoskeletal proteins are well conserved between animal and plant kingdoms; nevertheless, because plant cells exhibit major structural differences to animal cells, the question arises whether the plant cytoskeleton also responds to geometrical cues. Recent numerical simulations predicted that a geometry-based rule is sufficient to explain the microtubule (MT) organization observed in cells. Due to their high flexural rigidity and persistence length of the order of a few millimeters, MTs are rigid over cellular dimensions and are thus expected to align along their long axis if constrained in specific geometries. This hypothesis remains to be tested *in cellulo*. Here, we explore the relative contribution of geometry to the final organization of actin and MT cytoskeletons in single plant cells of *Arabidopsis thaliana*. We show that the cytoskeleton aligns with the long axis of the cells. We find that actin organization relies on MTs but not the opposite. We develop a model of self-organizing MTs in three dimensions, which predicts the importance of MT severing, which we confirm experimentally. This work is a first step toward assessing quantitatively how cellular geometry contributes to the control of cytoskeletal organization in living plant cells.

## Significance

The cytoskeleton, a network of polymers including microtubules and actin, supports many functions in cells. In plants, the cytoskeleton orientation is an important parameter dictating the direction of cell growth. While light, hormonal, or mechanical signals can affect the cytoskeleton organization, the role of cell geometry remains to be clarified. With a microwell-based approach, we confined plant cells lacking walls in different geometries and found that the cytoskeletons align with the long axis in cells in rectangular wells. Basic geometrical rules of the microtubules are computationally modeled in three dimensions and reveal the role of severing proteins in the shape response, which was observed experimentally. These findings demonstrate how cell geometry feeds back on cytoskeletal organization in plant cells.

How organisms achieve their specific forms and alter their growth patterns in response to environmental signals has been the subject of investigation for over a century (1). The importance of shape for specific biological functions has been shown across kingdoms (2–4). As an example, the aperture and closure mechanism of pores allowing gas exchange between plants and the environment rely on the shape of stomatal guard cells in leaves of flowering plants (3, 5, 6). Another example is that the elongation of cells promotes a prohealing phenotype in macrophages (4).

The cytoskeleton, an interconnected network of filamentous polymers such as actin and microtubules (MTs), plays a key role in establishing robust cell shape. In animal cells, the actomyosin cortex gives the cells their mechanical strength and acts in controlling their shapes (7–10). In plant cells, a cortical MT network, rather than actin, prescribes the shape of cells by guiding the synthesis of cellulose in the cell wall (11) and supports the mechanical strength of plant protoplasts (plant cells with their walls enzymatically removed) (12). In growing plant cells, the actin network is also involved in cell wall formation by being responsible for the global distribution of cellulose synthase complexes at the plasma membrane (13). The cytoskeleton is thus a major determinant of cell shape. In turn, cell geometry has been shown to impact cytoskeletal organization in animal cells (14–18). Moreover, a geometrical restriction is known to alter nuclear morphology and chromatin compaction (19, 20), highlighting the impact of cell geometry in modulating transcriptional response. In plants, several external factors have been shown to influence MT cytoskeletal organization such as light, hormones (21), and mechanical stresses (22–24). The analysis of the role of geometric cues in controlling cytoskeleton *in planta* is lacking and remains to be quantified. Cytoskeletal organization in animal and plant cell types is quite different. While the mechanical properties of animal cells and their shape integrity rely mainly on a dense network of cortical actin, interphase MTs are much less abundant and are involved in intracellular vesicle trafficking during interphase (25, 26). Conversely, plant cells display a dense network of cortical MTs that is involved in cell wall synthesis (11, 27). The actin cytoskeleton in plant cells is mostly found within more central regions of the cytoplasm, notably within cytoplasmic strands that cross the vacuole and connect to the nucleus (28). In addition, plant cells lack centrosomes, which leads to diffuse MT nucleation within the cell (*SI Appendix*, Fig. S1). Although cytoskeletal proteins are well conserved in eukaryotes, the differences in organization raise the question of whether cytoskeletal response to geometrical cues is conserved between plants and animals.

*In planta*, different MT organizations can be observed in cells with similar geometry within the same tissue, for example in epidermal regions within the shoot apical meristem or in different cell layers in the hypocotyl of *Arabidopsis thaliana* plants. Tissue shape-derived mechanical stresses were shown to play a dominant role in organizing the MTs in these cases (23, 24). Because of the coupling between mechanical stress and tissue geometry, the role of cell geometry alone on cytoskeletal organization can be difficult to estimate when working at the tissue level. Due to their high flexural rigidity and persistence length of the order of a few millimeters (29), MTs are rigid over cellular dimensions and can be expected to align along their long axes if constrained in specific geometries. However, many models predicted that MT–MT interactions influence the ability of alignment (30–34), and the alignment along the longest axis hypothesis remains to be tested *in cellulo*. Specifically, the severing of MTs at crossing sites by the protein katanin (35–38) has been proposed to promote MT alignment. Recently, an extensive theoretical study of the effect of severing of MTs highlighted the importance of taking into account this dynamical property in two-dimensional (2D) simulations (39). The MT organization that emerges from 3D simulations when incorporating the severing rule, and whether that corresponds with experimental observations, is not clear. Recent in vitro experiments with semiflexible filament solutions (40–43) and three-dimensional (3D) models in embryonic plant cells (44–46) predicted that a geometry-based rule is sufficient to explain the MT organization. However, this model has not been empirically tested.

It is known that actin organization is influenced by mechanical forces in animal cells (47–49), but how actin filaments respond to mechanical stresses has rarely been investigated in plants (50–52). Because the actin filaments and MTs have been shown to locate near the plasma membrane in plant cells, it has been suggested that the two networks interact (53). The specific interactions between actin and MTs are poorly understood, but one study demonstrated that actin filament reassembly depends on MTs (54), possibly via formin interactions (54–56). Whether actin filaments and MTs behave similarly or interact in the context of a response to geometrical or mechanical perturbation still remains to be established.

In this study, we developed an experimental approach to explore the contribution of geometry to the final organization of actin and MT cytoskeletons in isolated plant cells lacking cell walls. We extended a previously developed model of the 3D self-organizing MT network to predict the role of severing MTs on the shape response. We show that geometry alone is sufficient to explain the observed reorganization of MTs in rectangular shapes and that the geometry response is dependent on severing. In addition, studying actin and MTs in the same system allowed us to compare the organization of the two networks, and how they interact. We show that the actin response to shape change depends on the MT network but not vice versa.

## Results

**Fig. 1.**
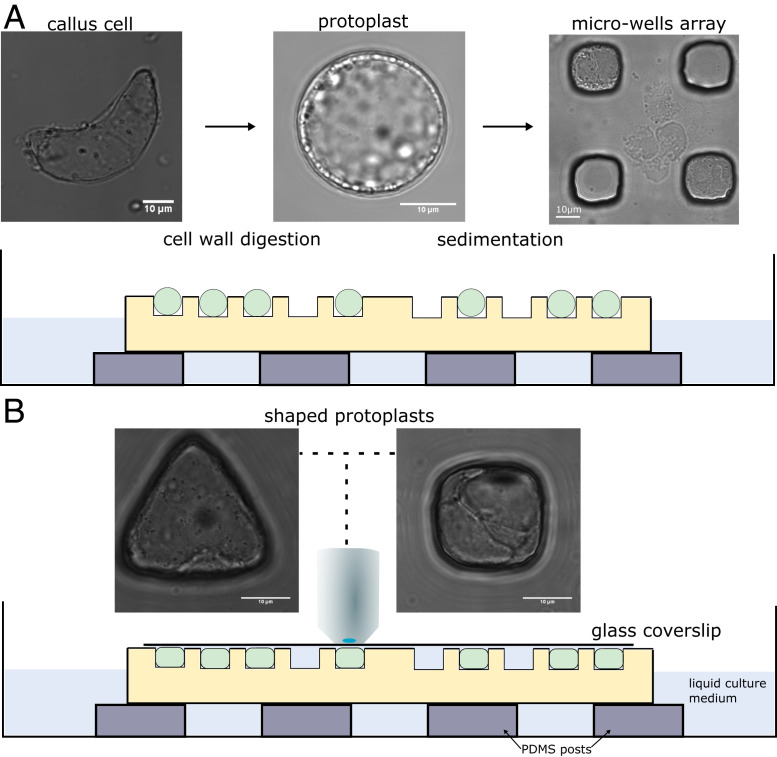
Description of the experimental setup used for the confinement of protoplasts in different geometries. (*A*) The cell walls of *Arabidopsis thaliana* root-derived callus cells were digested to make spherical protoplasts. Protoplasts were then plated on top of the microwell array. (*Left*) Bright-field picture of an isolated cell from callus with undefined geometry. (*Middle*) Bright-field picture of a freshly isolated protoplast with spherical geometry. (*Right*) Bright-field picture of protoplasts in square microwells after sedimentation. (*Bottom*) Illustration of microfluidic setup with cells loaded in wells. (*B*) Coverslips were carefully placed on top of the microwells, and the samples were imaged using an upright microscope. Bright-field pictures of protoplasts being shaped in microwells of various geometries (63× objective). All size standards are 10 µm.

To study how geometry affects cytoskeletal organization in plant cells, we developed a microwell-based approach allowing the control of the geometry of cells. The approach has been successfully used in the past to control, for example, the geometry of sea urchin embryos (57). The principle consists of confining single cells within microfabricated microwells of various geometries (Fig. 1 and *SI Appendix*, Fig. S2). Because we aim to analyze the effect of geometry independently from mechanical stresses in the wall and from tissue signals, plant cell walls were digested and single protoplasts were used for experiments. In order to be properly fitted into the microwells, the osmotic pressure of the protoplasts was reduced to 280 mOsm (Fig. 1 and *Materials and Methods*). In these conditions, the protoplasts were found to be regenerating their walls after 3 d in culture (*SI Appendix*, Fig. S3), suggesting that the culture medium allowed normal physiological functions in the protoplasts used in our study. We used green fluorescent protein fused to a MT-binding domain (MBD-GFP) (58, 59) and green fluorescent protein fused to an F-actin binding domain (FABD-GFP) (60) in the reporter lines for monitoring the MT network and the actin network, respectively. The microwells were cast from protoplast culture medium with 1.5% agarose (*Materials and Methods*) with wells of various shapes and a set of diameters ranging from 15 to 40 µm.

### MT Organization in Protoplasts Confined in Controlled Geometries.

**Fig. 2.**
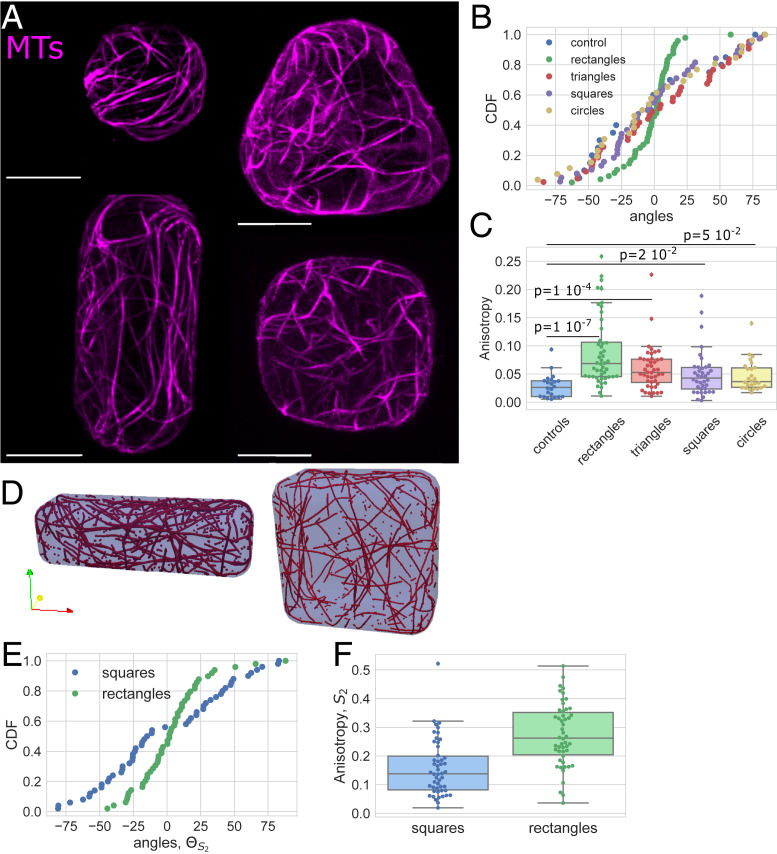
Effect of the shape on the MT network organization. (*A*) Protoplasts expressing MBD-GFP reporter confined in microwells of different geometries showing the effect of shape on the MT network organization. All size standards are 10 µm. (*B*) Cumulative distribution of the average angle of the MT network for the various geometries tested. Number of protoplasts *N* = 44 and number of repeats *n* = 12 for triangles, *N* = 48 and *n* = 10 for rectangles, *N* = 38 and *n* = 11 for squares, *N* = 26 and *n* = 7 for circles, and *N* = 22 and *n* = 4 for controls. (*C*) Boxplot of the anisotropy of the MT network in the different geometries tested. The number of protoplasts *N* and number of repeats *n* are the same as in *B*. (*D*) Example of the MT arrangement at the end of numerical simulations for a square domain and a rectangle domain. (*E*) Graph of the average angle of the MT network in rectangular and square shapes from numerical simulations of *t*_steps_ = 10,000 time steps, *R* = 50 repeats, and *P*_cross_ = 0.005 (with severing of MTs). (*F*) Anisotropy of the MT network for rectangular and square shapes in numerical simulations of *t*_steps_ = 10,000 time steps, *R* = 50 repeats, and *P*_cross_ = 0.005 (with severing of MTs).

**Fig. 3.**
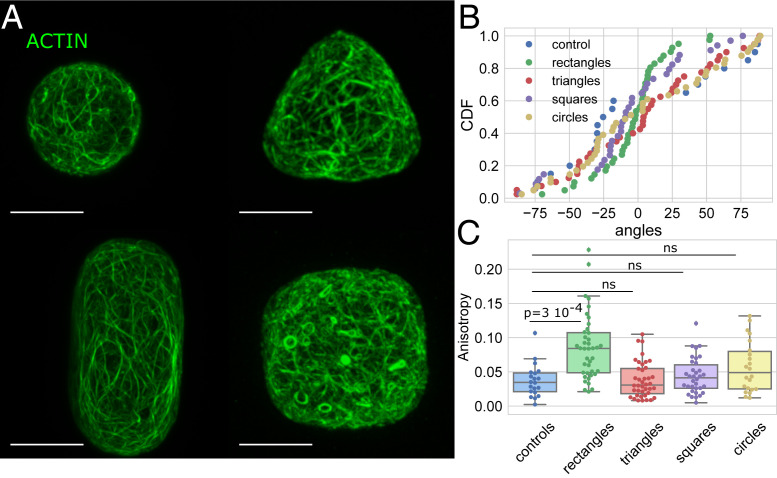
Effect of the shape on the actin network organization. (*A*) Protoplasts expressing FABD-GFP reporter confined in microwells of different geometries showing the effect of shape on the actin network organization in protoplasts. All size standards are 10 µm. (*B*) Cumulative distribution of the average angle of the actin network for the various geometries tested. Number of protoplasts *N* = 40 and number of repeats *n* = 11 for triangles, *N* = 40 and *n* = 12 for rectangles, *N* = 34 and *n* = 12 for squares, *N* = 22 and *n* = 3 for circles, *N* = 40 and *n* = 2 for controls. (*C*) Boxplot of the anisotropy of the actin network in the different geometries tested; the *P* values of KS test are provided as an indication, ns for values of *P* > 0.05. The number of protoplasts *N* and number of repeats *n* are the same as in *B*.

We first tested the effect of geometry on the organization of cytoskeletal networks in unperturbed protoplasts (61). For that purpose, we chose to focus on microwells with simple shapes, each with an in-plane geometry of a circle, square, triangle, or rectangle (Fig. 2 and *SI Appendix*, Fig. S2). The control shape corresponds to spherical unperturbed protoplasts embedded in low melting point agarose. Cells were observed between 30 min and 5 h after being fitted in the microwells. The average angle and the anisotropy of the MT (Fig. 2*A*) and actin (Fig. 3*A*) networks in protoplasts in those shapes were then quantified (*Materials and Methods*). For all of the shapes, the average orientation is defined relative to the *x* axis of the image, which is parallel to the long axis of the rectangular shapes and parallel to one of the sides for the other shapes.

We found that the average orientation of the MT network is not biased in circular, triangular, and square shapes (Fig. 2*B* and *SI Appendix*, Figs. S4 and S5). The distributions of the average angle showed no preferred orientation over all of the cells tested in these shapes. Moreover, the distributions are statistically comparable to uniform distributions (Fig. 2*B* and *SI Appendix*, Fig. S4). However, the anisotropy (measured as the difference of the eigenvalues of the nematic tensor calculated from the intensity gradients in individual cells; *SI Appendix*, *SI Materials and Methods*) of the MT network, in cells in triangular, square, and circular shapes is always significantly higher than the anisotropy of the MT network in spherical protoplasts, the control shapes (Fig. 2*C*; Kolmogorov–Smirnov [KS] *P* values of 10^−7^, 10^−4^, 0.02, or 0.05 for rectangular, triangular, squares, and circular shapes, respectively; *SI Appendix*, Fig. S6). This suggests that confinement alone is enough to influence the alignment of MTs.

The average orientation of the MT network was strongly biased toward the long axis in protoplasts in rectangular shapes (Fig. 2*B* and *SI Appendix*, Figs. S4 and S5). The cumulative distribution of the average angle for the MT network in protoplasts confined in elongated shapes shows an accumulation around the 0° angle (which corresponds to the long axis of the shape) (Fig. 2*B* and *SI Appendix*, Figs. S4 and S5; *P* value Kuiper = 10^−16^). The anisotropy of the MT network in elongated shapes is also significantly higher than for the control, suggesting that the MTs are better aligned to one another in the elongated shapes (Fig. 2*C*; *P* value, KS test = 10^−7^; *SI Appendix*, Fig. S6). These results in living single plant cells provide experimental evidence for the MT organization that was predicted in silico via numerical simulations (44) and in vitro (40, 62).

We extended a previously developed 3D model (44) by adding crossover severing to the MT interactions (*Materials and Methods* and *SI Appendix*, Fig. S7). Briefly, we modeled each MT as connected line segments with the MT growing, shrinking, and interacting with other MTs and the cell membrane, and included both bundling and induced catastrophe rules (*Materials and Methods* and *SI Appendix*, *SI Materials and Methods* and Fig. S7). MTs are not allowed to cross the boundary domain. When using parameter values extracted from experiments or previous modeling studies (*Materials and Methods* and *SI Appendix*, *SI Materials and Methods* and Table S1), the 3D model with severing leads to alignment of the MT network with the long axis in rectangular shapes (Fig. 2 *D*–*F*). Contrary to what was found before in square domains without severing of the MTs (44), the simulations with severing rules did not exhibit a preferred alignment along the diagonals of the square, in agreement with our experimental data (Fig. 2 *B* and *C*). In addition, both simulations and the experiments reveal that the anisotropy of the network is higher in rectangular domains than in square domains (Fig. 2 *C* and *F*). This suggests that geometry can be sufficient to organize the MTs in some living plant cells and that severing may play an important role. The simulations further predict that there is indeed a dependence of the anisotropy magnitude on the severing parameter (*SI Appendix*, Fig. S8).

### Actin Organization in Protoplasts Confined in Controlled Geometries.

We next quantitatively analyzed the actin organization (Fig. 3*A*). Our experiments suggest that the average orientation of the actin network is not influenced by geometry for cells in circular, triangular, and square shapes (Fig. 3*B* and *SI Appendix*, Fig. S4). Similar to MTs, the distributions of the average angle showed no preferred actin orientation in cells in these shapes and were comparable in uniformity to the distribution found in spherical protoplasts (the control shape; values of *P* > 0.05 with Kuiper’s test; *Materials and Methods*, Fig. 3*B*, and *SI Appendix*, Fig. S4). The anisotropy of the actin network in triangular, square, and circular shapes is not significantly different from the anisotropy of the actin network in spherical protoplasts (Fig. 3*C*; KS test gave values of *P* > 0.05; *SI Appendix*, Fig. S6). Similar to what was found for MTs, the average orientation of the actin network was found to be strongly biased toward the long axis in protoplasts in rectangular shapes (Fig. 3*B*). The cumulative distribution of the average angle for the actin network in protoplasts confined in rectangular shapes shows an accumulation around the 0° angle, which corresponds to the long axis of the rectangle (Fig. 3*B* and *SI Appendix*, Fig. S4; *P* value Kuiper = 8 × 10^−6^). Moreover, the anisotropy of the actin network in rectangular shapes is significantly higher than for the control shape, suggesting that the actin filaments are better aligned to one another in the rectangular shapes (Fig. 3*C*; *P* value KS = 3 × 10^−4^; *SI Appendix*, Fig. S6). In the conditions of our experiments, actin follows cell shape as in vitro (41, 42) indicating that intrinsic mechanical properties of actin filaments could dictate their orientation.

### Severing of Microtubules by Katanin Is Necessary for the MT Response to Shape.

**Fig. 4.**
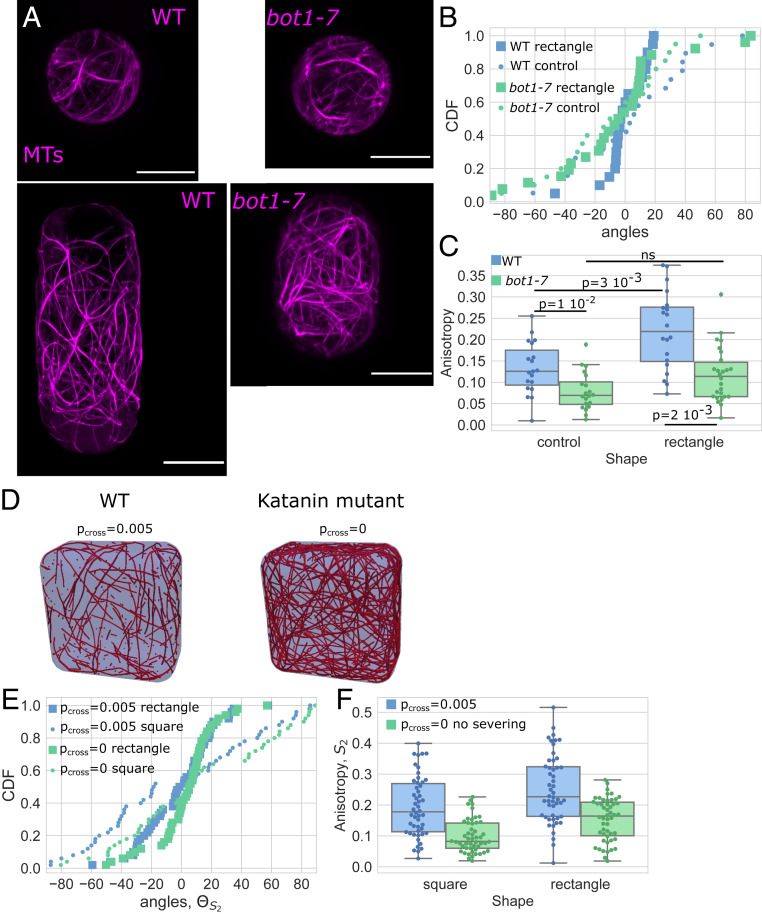
Shape response in protoplasts with genetically disturbed MTs dynamics. (*A*) Protoplasts expressing MBD-GFP reporter confined in microwells showing the effect of shape on the MT network organization in wild-type protoplasts (*Left*) and protoplasts generated from calli in the *bot1-7* mutation background (*Right*). All size standards are 10 µm. (*B*) Cumulative distributions of the average angle of the MT network of wild-type (blue) and *bot1-7* (green) protoplasts in rectangular microwells (square markers) and in control-shaped (circle markers). Number of protoplasts *N* = 19 and number of repeats *n* = 3 for wild-type protoplasts in control shapes, *N* = 20 and *n* = 3 for wild-type protoplasts in rectangular shapes, *N* = 20 and *n* = 1 for *bot1-7* protoplasts in control shapes, and *N* = 26 and *n* = 1 for *bot1-7* protoplasts in rectangular shapes. (*C*) Boxplot comparing the anisotropy of the MT networks of wild-type (blue) and *bot1-7* (green) protoplasts. The number of protoplasts *N* and number of repeats *n* are the same as in *B*. (*D*) Example of the MT arrangement from numerical simulations with (*Left*) and without (*Right*) crossover severing. (*E*) Graph of the average angle of the MT network for rectangular and square shapes in numerical simulations of *t*_steps_ = 3,000 time steps with (*P*_cross_ = 0.005) and without (*P*_cross_ = 0) crossover severing for each case averaged over *R* = 50 simulations. (*F*) Anisotropy of the MT network for rectangular and square shapes in numerical simulations of *t*_steps_ = 3,000 time steps with (*P*_cross_ = 0.005) and without (*P*_cross_ = 0) crossover severing for each case averaged over *R* = 50 simulations.

To investigate experimentally whether the severing activity is required for the MT response, we performed experiments on protoplasts expressing the MBD-GFP reporter in the katanin mutant background *bot1-7* (Fig. 4*A*). Katanin is a protein known to bind MTs and promotes the severing of MTs at crossover sites (38, 63). We focus on protoplasts in control and rectangular shapes as these exhibited the most significant effect in the overall orientation and alignment of the MT network. We found that the average angle of the MT network was still distributed following the long axis of the shape but that the number of cells with a transverse organization was increased in comparison to wild type. For the wild type, almost all cells exhibited an average angle between −20 and 20° (Fig. 4*B*; *n* = 19/20), whereas in the mutant more than one-third of the cells exhibited an average angle higher than 20° (*n* = 10/26) and some cells had a dominating transverse array of MTs (*n* = 5/26) (Fig. 4*B* and *SI Appendix*, Fig. S9). Moreover, the distribution of the average angle for the MTs in the mutant protoplasts in rectangular shapes was no longer different compared to a uniform distribution (Fig. 4*B* and *SI Appendix*, Fig. S9; Kuiper test gives a value of *P* > 0.05).

In addition, in the *bot1-7* mutant, the anisotropy of the MT network was smaller than in wild-type spherical protoplasts (Fig. 4*C*). This defect in anisotropy of the network for this mutant is already known from *in planta* studies (64, 65). In protoplasts from *bot1-7* mutant cells, the anisotropy of the MT network was not significantly higher in the protoplasts plated in rectangular wells when compared to spherical protoplasts (Fig. 4*C* and *SI Appendix*, Fig. S10). While the rectangular shape created a bias toward better alignment of MTs in the wild type, this bias was reduced in the absence of severing by katanin. Our experiments suggest that loss of katanin function makes the MT cytoskeleton less responsive to geometrical cues.

Next, we used simulations to test how tuning crossover severing (representing severing by katanin) affects MT alignment in different geometries (Fig. 4 *D*–*F*). Our computational results demonstrate that removing severing at MT crossovers induces lower anisotropy in square and rectangular domains (Fig. 4*F* and *SI Appendix*, Fig. S8). The change in anisotropy induced by removing the severing rule (i.e., equivalent to the katanin mutant in the experiments) is robust across parameters (*SI Appendix*, Fig. S11). This corroborates our experimental findings that katanin influences MT organization in response to shape change.

### Actin Polymerization Defect Impairs the Organization of Actin in Response to Shape.

**Fig. 5.**
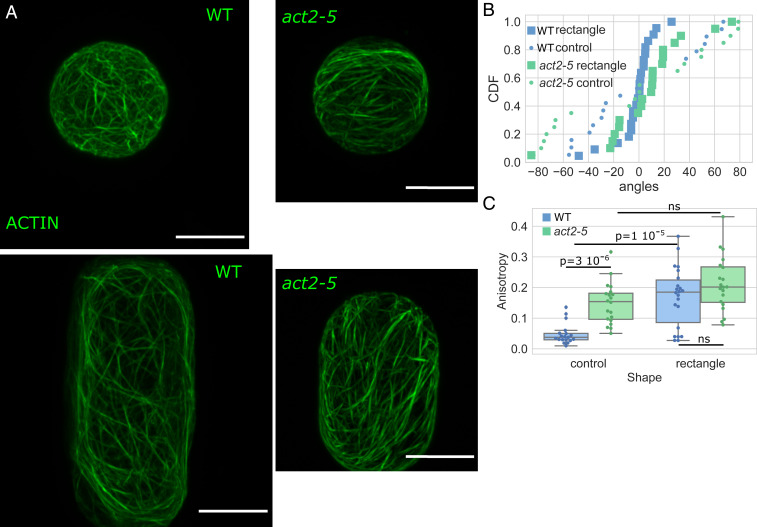
Shape response in protoplasts with genetically disturbed actin dynamics. (*A*) Protoplasts expressing FABD-GFP reporter confined in microwells showing the effect of shape on the actin network organization in wild-type protoplasts (*Left*) and protoplasts generated from calli in the *act2-5* mutation background (*Right*). All size standards are 10 µm. (*B*) Cumulative distributions of the average angle of the actin networks of wild-type (blue) and *act2-5* (green) protoplasts in rectangular microwells (square markers) and in control shape (circle markers). Number of protoplasts *N* = 19 and number of repeat *n* = 1 for wild-type protoplasts in control shapes, *N* = 22 and *n* = 4 for wild-type protoplasts in rectangular shapes, *N* = 20 and *n* = 2 for *act2-5* protoplasts in control shapes, and *N* = 20 and *n* = 2 for *act2-5* protoplasts in rectangular shapes. (*C*) Boxplot comparing the anisotropy of the actin networks of wild-type (blue) and *act2-5* (green) protoplasts. The number of protoplasts *N* and number of repeats *n* are the same as in *B*.

We next tried to challenge the robustness of the actin response to shape by testing whether an alteration in actin polymerization affected actin organization in response to shape. The *act2* mutant is known to show disrupted actin polymerization and cells in the mutant exhibit shorter actin filaments (66, 67). The average angle for the actin network was found to be aligned to the main axis of the shape for a majority of *act2-5* protoplasts confined in rectangular shapes and the average angle was found to be uniformly distributed for spherical *act2-5* protoplasts, similarly to wild-type protoplasts (Fig. 5 *A* and *B* and *SI Appendix*, Fig. S9). The distribution of the anisotropy of the actin network for *act2-5* protoplasts in the rectangular shape is not significantly different from the distribution in spherical *act2-5* protoplasts (value of *P* = 0.2). Moreover, we found that actin in *act2-5* spherical protoplasts exhibited higher anisotropy than in the wild-type spherical protoplasts (Fig. 5*C* and *SI Appendix*, Fig. S10). Altogether, our results suggest that while the long axis is selected in the mutant, actin polymerization and filament length are important for the magnitude of the alignment of the cell shape response.

### Actin Organization in Protoplasts Confined in an Elongated Geometry Relies on the MT Network but Not Vice Versa.

**Fig. 6.**
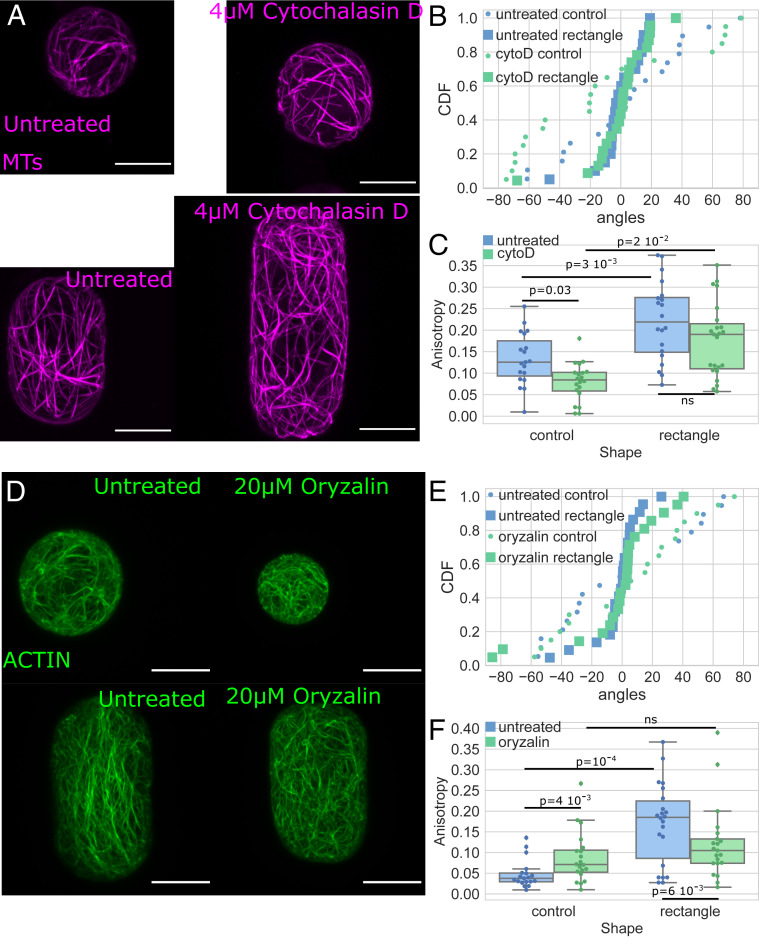
Shape response in protoplasts treated with cytoskeletal drugs. (*A*) MTs: protoplasts expressing MBD-GFP reporter confined in microwells showing the effect of shape on the MT network organization in untreated protoplasts (*Left*) and protoplasts treated with cytochalasin D (*Right*). All size standards are 10 µm. (*B*) Cumulative distributions of the average angle of the MT networks of untreated protoplasts (blue) and protoplasts treated with 4 μM cytochalasin D (green) in rectangular microwells (square markers) and in control shape (circle markers). Number of protoplasts *N* = 19 and number of repeat *n* = 3 for untreated protoplasts in control shapes, *N* = 20 and *n* = 3 for untreated protoplasts in rectangular shapes, *N* = 20 and *n* = 2 for treated protoplasts in control shapes, and *N* = 23 and *n* = 1 for treated protoplasts in rectangular shapes. (*C*) Boxplot comparing the anisotropy of the MT networks of untreated protoplasts (blue) and protoplasts treated with 4 μM cytochalasin D (green). The number of protoplasts *N* and number of repeats *n* are the same as in *B*. (*D*) Actin: protoplasts expressing FABD-GFP reporter confined in microwells showing the effect of shape on the actin network organization in untreated protoplasts (*Left*) and protoplasts treated with oryzalin (*Right*). All size standards are 10 µm. (*E*) Cumulative distributions of the average angle of the actin networks of untreated protoplasts (blue) and protoplasts treated with 20 μM oryzalin (green) in rectangular microwells (square markers) and in control-shaped (circle markers). Number of protoplasts *N* = 19 and number of repeat *n* = 1 for untreated protoplasts in control shapes, *N* = 22 and *n* = 4 for untreated protoplasts in rectangular shapes, *N* = 20 and *n* = 2 for treated protoplasts in control shapes, and *N* = 21 and *n* = 2 for treated protoplasts in rectangular shapes. (*F*) Boxplot comparing the anisotropy of the actin networks of untreated protoplasts (blue) and protoplasts treated with 20 μM oryzalin (green). The number of protoplasts *N* and number of repeats *n* are the same as in *E*.

Next, to compare better the actin and MT network organizations, we did an independent set of experiments with higher imaging resolution, focusing on rectangular shapes as those produced the greatest effect (Figs. 4*A*, 5*A*, and 6 *A* and *D* and *SI Appendix*, Figs. S12 and S13). This set of experiments resulted in an overall increase in anisotropy values, due to reduced signal-to-noise ratio, yet showed a similar trend as the previous experiments. We found that the anisotropy is always higher for the MT network than for the actin network (*SI Appendix*, Figs. S12 and S13), which may reflect the different rigidity of MT and actin filaments (29).

Since our results suggest that actin filaments and MTs organize similarly in rectangular shapes, we next asked whether the actin and MT networks interact with each other in isolated plant protoplasts under geometrical constraints. To investigate this, we first studied the organization of the MT network when actin was depolymerized with cytochalasin D (protoplasts expressing the MBD-GFP reporter were treated for 1 h with 4 μM CytoD) (Fig. 6*A* and *SI Appendix*, Figs. S12–S14). In this condition, the average angle of the MT network in protoplasts in the elongated shape was aligned with the long axis of the shape, meaning that depolymerization of the actin has no effect on the reorganization of MTs upon shape change in terms of preferred angle (Fig. 6*B* and *SI Appendix*, Figs. S12–S14). The anisotropy of the MT network still increased in protoplasts plated in elongated shapes as compared to spherical protoplasts (Fig. 6*C*; value of *P* = 0.02). Thus, the disruption of the actin network did not have a significant impact on the response of MTs to shape in the conditions of our experiments.

We then studied the organization of the actin network when the MTs were depolymerized with oryzalin (20 µM for 30 min; Fig. 6*D* and *SI Appendix*, Figs. S12–S14). In this condition, the average angle of the actin network in protoplasts in the elongated shape was still aligned with the long axis of the shape, meaning that absence of MTs had no significant effect on the orientation of actin after cell shape change (Fig. 6*E* and *SI Appendix*, Figs. S12–S14). In contrast to the untreated case, for protoplasts treated with oryzalin, the anisotropy of the actin network was not significantly higher in rectangular shapes than in control (Fig. 6*F*; see also Fig. 3*C*). This indicates that the MT alignment is required for the actin filaments to align well to one another in response to shape. Our findings suggest that the actin organization in response to shape change relies on the MT network while a dependence of MTs on actin was not observed.

## Conclusion/Discussion

In this study, we quantified cytoskeletal response to cell shape. By working with isolated protoplasts with no wall, the effects of the tension borne by the cell wall and possible tissue signals were eliminated. Such signals include stress and strain mechanical signaling resulting from tissue shape and turgor pressure, and intercellular signaling. In these conditions, we expect to be able to study the effect of geometry alone. We show that MTs and actin filaments are sensitive to cell geometry in living isolated plant cells and that they align predominantly along the long axis of cells. A model based on basic MT interaction rules and on intrinsic mechanical properties of MTs is sufficient to explain our experimental quantification. Therefore, at the single-cell level, geometry is a contributor to cytoskeletal organization in the plant cells we tested.

In rectangular shapes, both actin and MTs were found to be aligned along the long axis and the anisotropies of the networks were always higher than in spherical protoplasts. This is similar to what was found in vitro (40–42, 62), suggesting that the organization emerges from intrinsic mechanical and interaction properties of the network, a self-organizing process. In addition, we found that confinement alone is enough to influence the alignment of MTs. This was also observed in vitro when MTs were allowed to organize freely in droplets of different sizes (43). The alignment found in our experiments and in our simulations is a statistical tendency and does not exclude a minority of MTs in any direction. For triangular and square shapes, while we did not see a significant statistical bias of the shape on the organization, we found that for a small proportion of cells (5/30; *SI Appendix*, Fig. S5) MTs were aligned along the edges of triangular shapes or presented a weak bias toward the diagonals of square shapes (13/38 cells; *SI Appendix*, Fig. S5). This represents the longest distance across the polygon for these shapes. Contrary to what was found in animal cells for actin (14, 16) and predicted with numerical simulations of self-organizing MTs (44), we find only a weak bias toward diagonals in our experiments and simulations in the square shapes.

Studies on animal cells have shown the impact of geometry on the organization of actin and MTs (14, 16, 18, 68, 69). When cells are allowed to spread on 2D patterns of various geometries, actin exhibits different organizations, with alignment along the long axis of elongated shapes or along the diagonal of square shapes (14, 15). It is believed that through focal adhesions, tension builds up and actin reorganizes accordingly along tension lines (70). When human embryonic stem cells spread into 3D microniches of different shapes, geometry influenced the actin organization (16, 17). Cortical areas of animal and plant cells are believed to exhibit different distributions of actin and MTs. In addition, when a plant cell is turgid, the vacuole may restrict the cytoplasm to a thin compartment, which could alter cytoplasmic mechanical properties [like viscosity and jamming (71)] and affect cytoskeletal regulation and reorganization following geometry changes. Despite these major structural differences, plant protoplasts and animal cells share common mechanical properties and exhibit similar stiffness (12). We found that plant cells exhibited similar cytoskeletal organization to animal cells in rectangular shapes, suggesting that at least some of the processes driving the response to geometry change could be universal.

In vivo, transverse organization of MTs is often observed in elongated plant cells, an orientation we did not observe in our system using wild-type protoplasts in rectangular shapes. In plant cells, the wall provides mechanical strength and physical constraint. At the same time, the wall is the domain of mechanical and intercellular signaling. In our experiments, the microwells provided the physical strength, while the tissue-scale mechanical and intercellular signals were absent. Thus, we suggest that the observed MT organization is the default one where the geometry dictates filament alignment due to steric interactions and that other organizations observed *in planta* would be due to intercellular signaling (like hormones, light, ions transport, or tissue shape-derived mechanical stress). Moreover, it has been shown in numerical simulations that a small bias in the growth direction of MTs is sufficient to alter this longitudinal organization into a transverse one (44). Similarly, while both transverse and longitudinal populations can be found in numerical simulations and in experiments, and while MTs are allowed to go around the membrane *in cellulo* and in our simulations, the probability of catastrophe at the edges of the domain was shown to create a bias toward one orientation (45). This suggests that the geometry of the cells can influence cytoskeletal organization *in planta*. Thus, steric constraint due to the bending rigidity of MTs prevails in the absence of other biases such as mechanical stress or protein interaction. In vitro, an external geometry can strongly influence the spatial organization of semiflexible filaments if the dimension of the confinement is comparable to the persistence length of the filaments. For example, actin filaments align parallel to the long walls of microchambers as soon as the geometrical aspect ratio of the chamber is greater than 1.5, which indicates that the organization of the filaments is dominated by geometrical boundary interactions (42). Our data shows that MTs align with the long axis of rectangular shapes for any aspect ratio above 1.2 (*SI Appendix*, Fig. S15). This further supports the view that geometry influences the cytoskeletal organization in our system.

This study provides evidence that actin filaments and MTs interact in the context of a response to geometrical perturbation. *In planta*, it was already reported that actin distribution becomes more irregular when the MTs are depolymerized (72) and actin–MT interactions were previously suggested (54–56). Comparing actin and MT organization in the same system revealed that the anisotropy of the actin network is always smaller than for the MTs. This might be linked to the different nature of the filaments composing each network (MT being an association of dimers organized in tubes and actin being a linear composition of monomers). In vitro, while the MTs are rather stiff rods, the flexural rigidity of actin filaments is 1,000 times smaller than for MTs (29). This could also explain why the actin alignment is altered when MTs are depolymerized, while the overall orientation is not. In the absence of interaction with MTs, actin could more freely explore the space, leading to an apparently less organized network while the overall orientation is conserved in rectangular shapes. We do not exclude that MT alignment could be altered under actin depolymerization, but the effect observed was lower than the effect of MTs on actin alignment. In plant cells, because MTs are stiffer filaments, they might have a dominant role in the cytoskeletal organization in response to cell shape. Conversely, in animal cells, because of its contractile properties and its link to adhesive complexes, the actin network is in the front line of mechanical and shape change responses. However, the coupling between the MT and the actin networks is revealed in animal cells during division; the tension in asters of MTs, which further align cell division according to shape, depends on the actin network (73).

Our approach is sufficient to detect defects in the organization of the cytoskeletal network in mutants affecting cytoskeletal dynamics. The cell geometry could not fully rescue the defect of anisotropy in the MT network in protoplasts of katanin mutants. It was already shown that a katanin mutant responds less to changes in mechanical stress (35, 74) and that MT reorientation triggered by blue light requires the action of katanin proteins (63). In addition, our experiments suggest that a katanin mutant is less responsive to geometrical cues. The previous model of dynamical MTs predicted no influence of cell shape on the anisotropy of the network (44), but experiments suggested the opposite with higher anisotropy for rectangular shapes. By refining the model and adding the severing rule accounting for katanin action, the simulations recapitulated the effect of shape change on the anisotropy of the MT network. In summary, we propose that in plant protoplasts of *A. thaliana* and in the conditions of our experiments, the MTs self-organize and severing by katanin allows a strong response to geometry change. As our 3D simulations of the MT network support the experimental observations, we conclude that geometry is sufficient to explain the specific organization observed.

## Materials and Methods

### Plant Growth Conditions and Protoplasts Generation.

We used green fluorescent protein fused to a MT binding domain (MBD-GFP) (58, 59) and green fluorescent protein fused to an F-actin binding domain (FABD-GFP) (60) reporter lines. All *A. thaliana* seeds were surface sterilized by ethanol (70%), stratified at 4 °C for 2 d, and grown vertically on plates containing Murashige and Skoog medium supplemented with half sucrose under continuous light. Calli were prepared from 2-wk-old seedlings. Protoplasts were obtained from calli as previously described (12) by a combination of cell wall degradation and hypoosmotic shock (*SI Appendix*, *SI Materials and Methods*).

### Drug Treatments.

For drug treatments, final concentrations of 4 µM Cytochalasin D diluted in 100% DMSO (Sigma) or 20 µM oryzalin diluted in DMSO were added to protoplasts for 30 min prior to observation (12). Drug-treated protoplasts were imaged in the presence of the drugs. The efficiency of drug treatment is described in *SI Appendix*, Fig. S14.

### Experimental Device, Image Acquisition, and Image Analysis.

Microchambers were fabricated following standard microfabrication techniques (75) (*SI Appendix*, *SI Materials and Methods*). Protoplasts lodged in microchambers were imaged with a Zeiss 780 or Zeiss 880 Airyscan confocal laser-scanning microscope with a 63× oil objective, and *z* stacks of cells with 0.18-μm intervals were obtained for 3D reconstruction. Zen 2.3 and ImageJ (76) (https://imagej.nih.gov/ij/) software were used to process images. *Z* stacks were projected on one plane with maximum intensity projection to create 2D images. Fibriltool (77) was used to quantify the organization of the cytoskeletal networks.

### Statistical Analysis.

For anisotropy comparison, two-sample KS tests were performed (and Student *t* tests for the computational parameter sweep comparisons in *SI Appendix*, Figs. S8 and S11). We used a bootstrap method to estimate the effect of the sample size. Kuiper tests were run on the angles dataset. Uniform distributions of the same number of angles as in the compared dataset were generated, and a two-sample Kuiper test was run (values of *P* higher than 0.05 were considered not significant).

### Numerical Simulation Description.

The 3D numerical model used is an adaptation of that developed in ref. 44 (*SI Appendix*, *SI Materials and Methods*, Table S1, and Fig. S7), where individual MTs grow, shrink, and interact with each other and the cell membrane. Primarily, we have extended the MT interactions. If a MT plus end gets close to another MT, and the interaction angle is below a threshold α, the MT bundles, by changing its growth direction to match (44). If the interaction angle is larger than the threshold, either an induced catastrophe occurs with probability *P*_cat_ (changing the MT from growing to shrinking) or the MT continues unimpeded growth, crossing the neighboring MT. The crossing MT can be severed at the crossover point with a probability *P*_cross_ at all later time steps. Severing breaks the MT into two at the cut element, with both MTs shrinking from their cut ends (*SI Appendix*, Fig. S7). We take *P*_cross_ = 0.005 for our default with-severing (wild-type) simulations, and *P*_cross_ = 0 for our default no-severing (katanin mutant) simulations (although a wider sweep of *P*_cross_ is shown in *SI Appendix*, Fig. S8). Parameter values are discussed in *SI Appendix*, Table S1 and *SI Materials and Methods*.

We compute MT anisotropy and the orientation of alignment in 2D on opposite faces of the domain using the $S_{2}$ order parameter and directional parameter $\Theta_{S_{2}}$ (*SI Appendix*, *SI Materials and Methods*).

The MT simulations were implemented in C++ with anisotropy calculations performed in Python on the output vtk files.

### Data Availability.

Original confocal data are available via the University of Cambridge Research Data Repository (https://doi.org/10.17863/CAM.51754); data for plotting the figures as well as code used for the analysis are available in the Sainsbury Laboratory GitLab repository (https://gitlab.com/slcu/teamhj/publications/durand_etal_2019). The 3D MT simulations were run using Tubulaton (https://gitlab.com/slcu/teamhj/Tubulaton).

## Supplementary Material

Supplementary File
