# Supplementary material for: Cytoskeletal organization in isolated plant cells under geometry control
Source: Proc Natl Acad Sci U S A. 2020 Jul 8;117(29):17399–408. doi: 10.1073/pnas.2003184117 (PMC7382239; doi:10.1073/pnas.2003184117)
Supplement: Supplementary File [file pnas.2003184117.sapp.pdf]

Supplementary Information

Cytoskeletal organisation in isolated plant cells under geometry control.

Pauline Durand-Smet[a], Tamsin A. Spelman[a], Elliot M. Meyerowitz[b,c,*], Henrik Jönsson[a,d,e,*]

a The Sainsbury Laboratory, University of Cambridge, Cambridge, United Kingdom

b Division of Biology and Biological Engineering, California Institute of Technology, Pasadena, USA

c Howard Hughes Medical Institute, California Institute of Technology, Pasadena, USA

d Department of Applied Mathematics and Theoretical Physics, University of Cambridge, Cambridge, United Kingdom

e Department of Astronomy and Theoretical Physics, Computational Biology and Biological Physics, Lund University, Lund, Sweden

*Henrik.Jonsson@slcu.cam.ac.uk

* meyerow@caltech.edu

Supplementary Figures

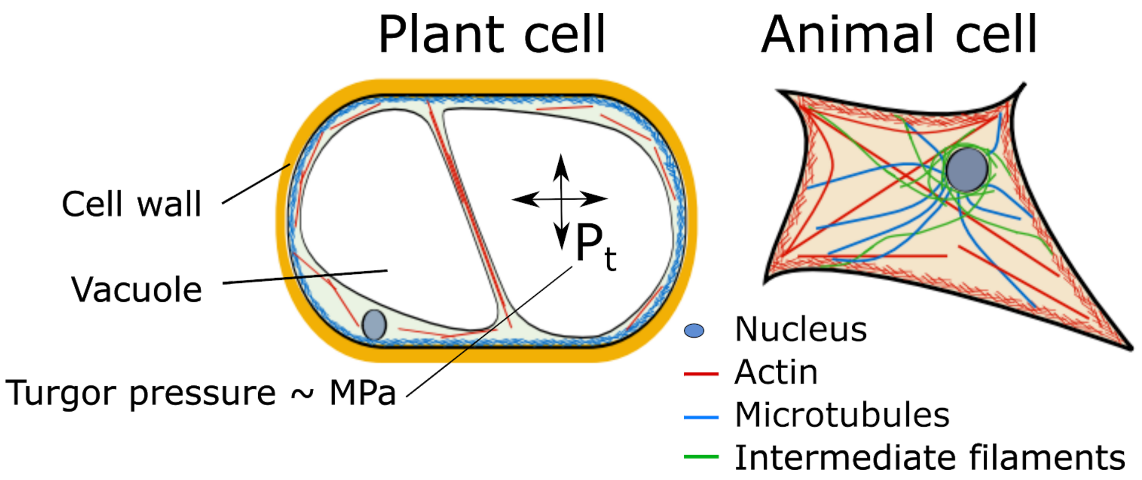

Figure S1: Drawing representing the structural elements of a typical plant cell and an animal cell.

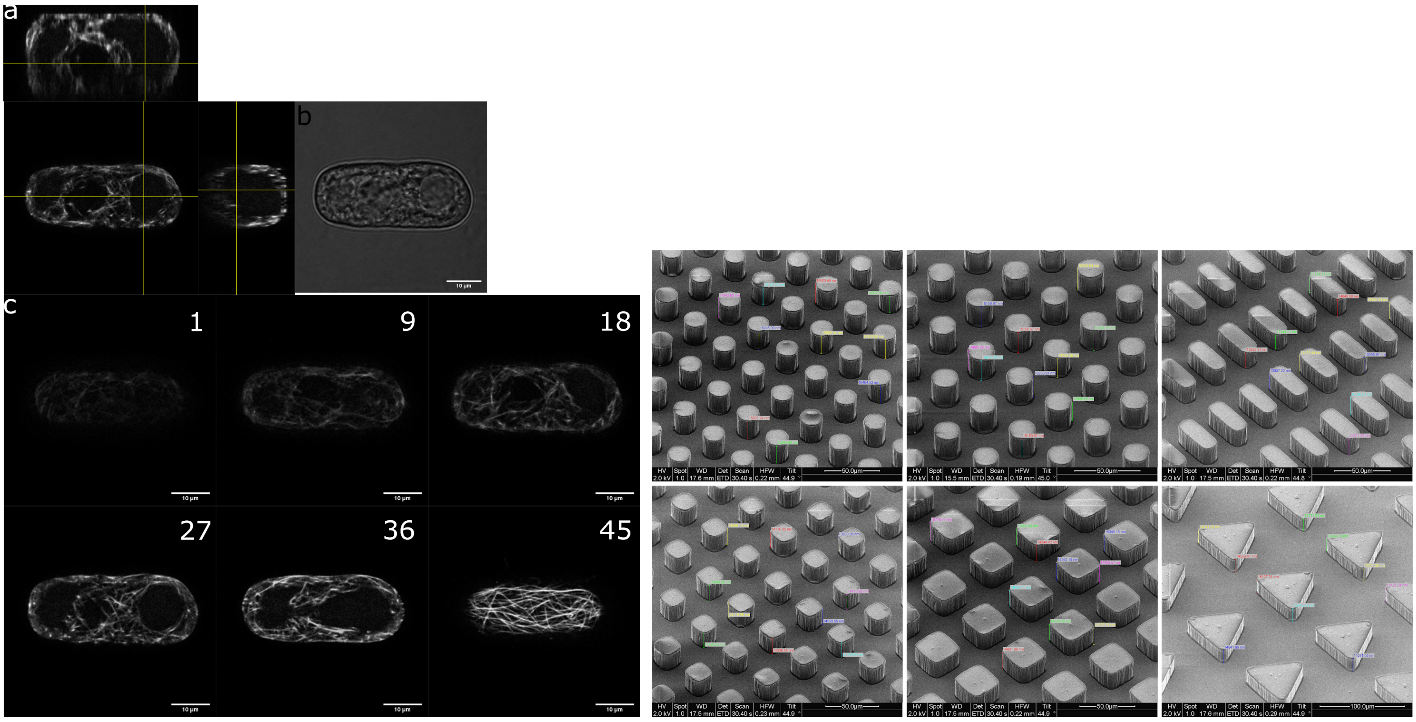

Figure S2: (a) orthogonal views of a protoplast expressing MBD-GFP reporter in a rectangular micro-well, scale bars are 10µm. The number at the top of each picture is the z focus plane (representative of the depth). (b) bright field picture of the same protoplast. (c) different focus planes of the same protoplast. (d) Scanning Electron Microscopy (SEM) pictures of silicon masters, with SU8 photoresist features, used as moulds for micro-chamber fabrication. The features are made of SU8-2025, an epoxy-based photoresist designed for micromachining (MicroChem) and the real average height is 20±0.3µm.(real height= height/sinα with α the tilt angle of the sample holder in the SEM, 44 degrees in the above pictures).

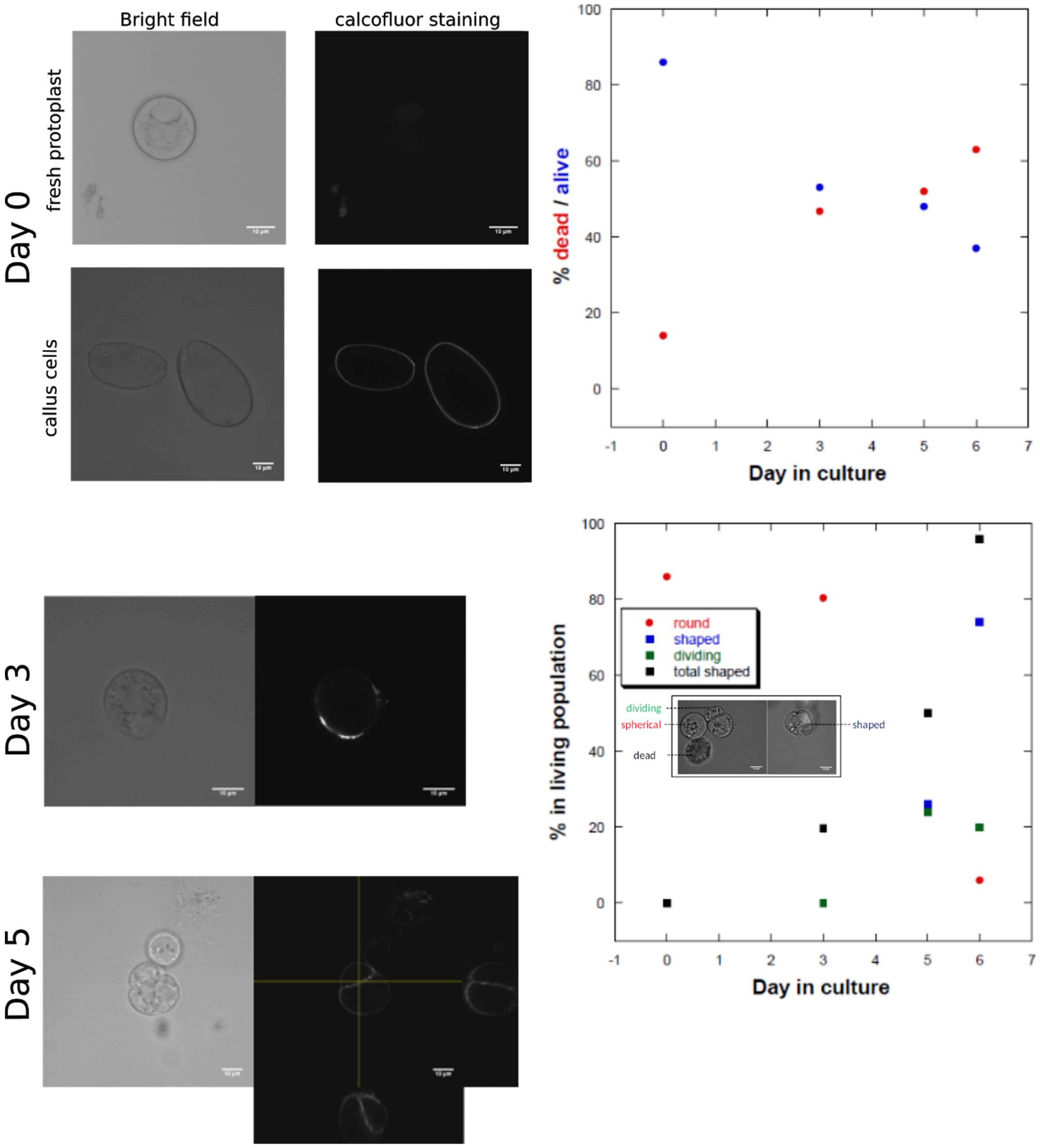

Figure S3: left: Calcofluor cell wall staining (0.01% CW) in fresh protoplast and callus cells. Fresh protoplasts do not exhibit cellulose fluorescence, in contrast to callus cells where an accumulation of cellulose can be observed in the conditions of staining. After 3 days and 5 days in culture, new cell walls start appearing in the protoplast culture, and some dividing cells can be observed, showing that the medium chosen allows protoplasts to survive for several days. The graphics on the right show the percentage of living protoplasts in the culture as a function of days in culture. While the living protoplast population decreases and reaches 40%, the number of protoplasts with new cell walls (shaped) or dividing in that population increases (bottom panel). For this regeneration and survival assay, protoplasts were cultivated in 5cm Petri dishes, plated at a density of 1.5 x10^6 cells/ml in the culture medium described in Materials and Methods. A counting of living, dead and shaped, dividing or round cells was performed with a hemocytometer.

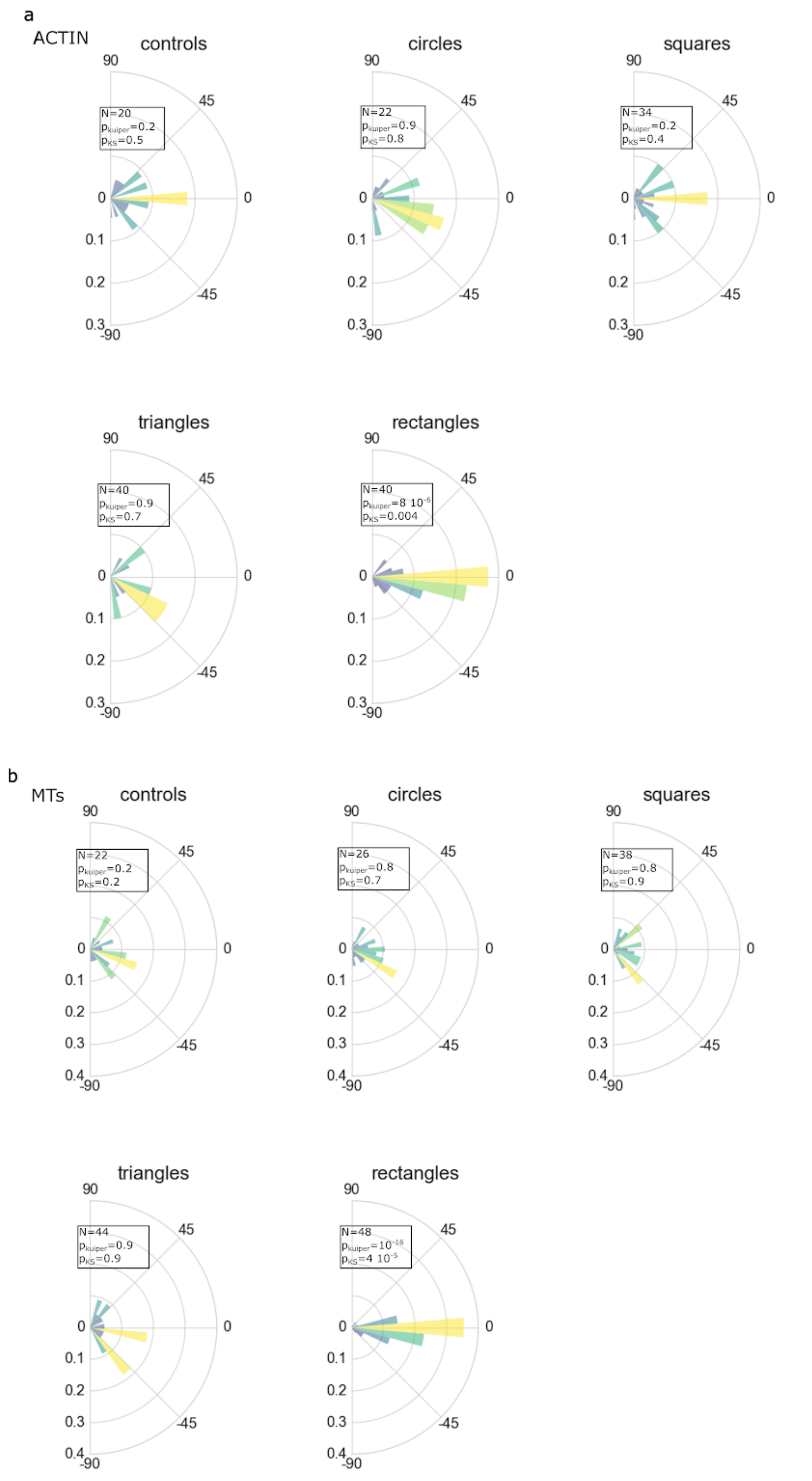

Figure S4: (a) Angular histograms of the average angle of the actin network in protoplasts in different geometries.
(b) Angular histograms of the average angle of the microtubule network in protoplasts in different geometries. p_{kuiper} and p_{KS} are the p-values returned by Kuiper test and Kolmogorov-Smirnov test where the average angle distributions are tested against uniform distributions generated with the same sample size.

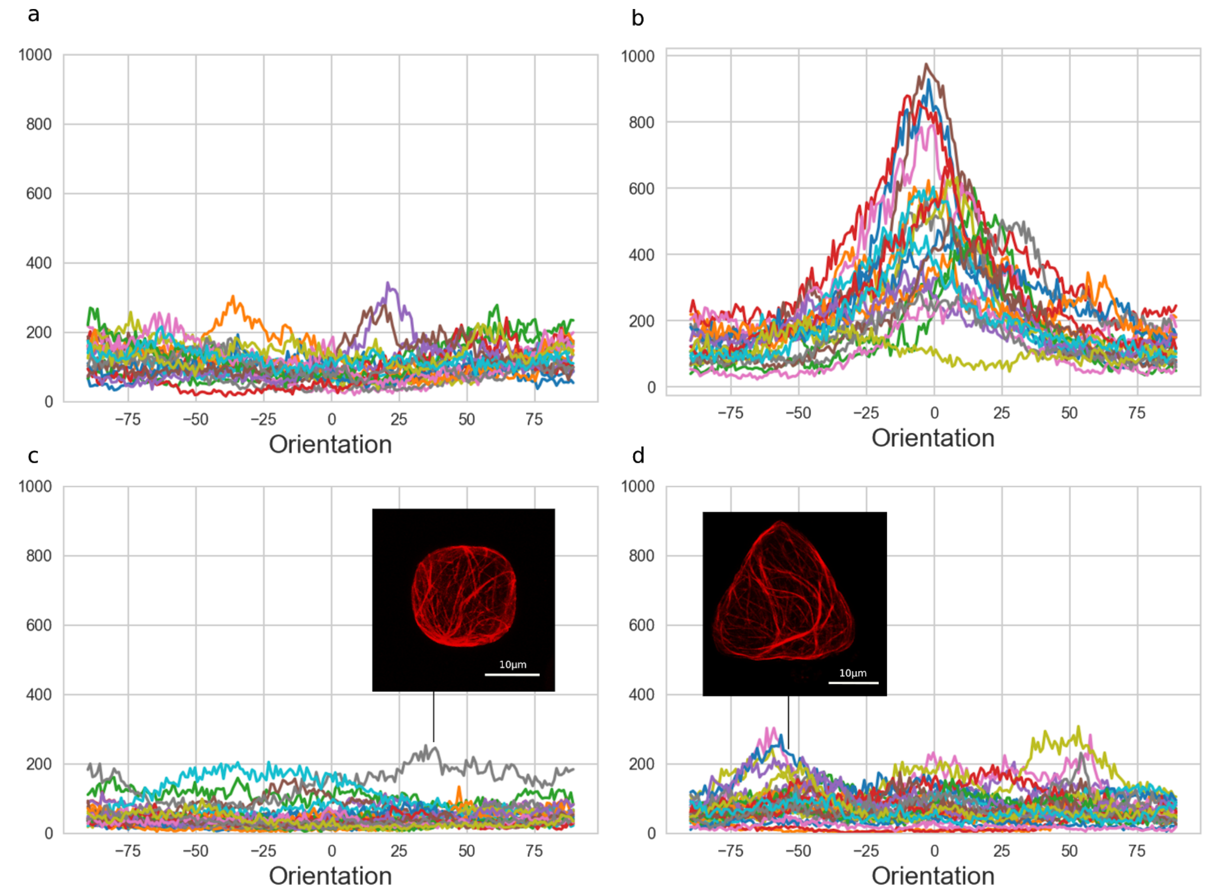

Figure S5: Angle distributions of microtubules of all cells in (a) control shapes (b) rectangular shapes (c) cubic shapes and (d) triangular shapes. The distributions were generated with OrientationJ, a Fiji plugin (http://bigwww.epfl.ch/demo/orientation/)(1) a local window size of 2 pixels and a cubic spline gradient were chosen to run the analysis for the orientation of the fibres.

(c) The insert picture shows the microtubule network of a protoplast in a cubic micro-well where the microtubules were organised along the diagonals (6/38 protoplasts presented such an organisation).

(d) The insert picture shows the microtubule network of a protoplast in a triangular micro-well where the microtubules were organised along the edges (5/30 protoplasts presented such an organisation).

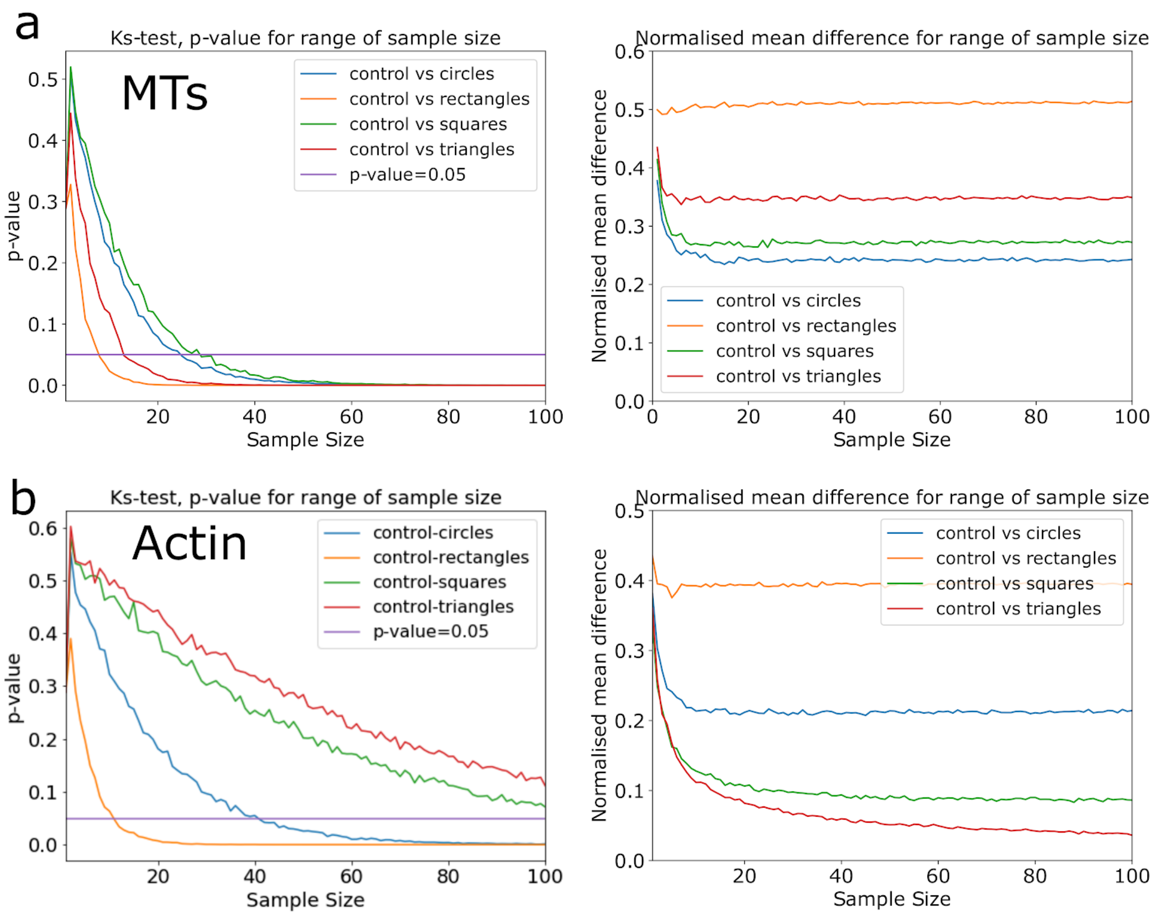

Figure S6: (a) Left: p-values obtained for KS tests of 1000 bootstrap repetitions of the anisotropy data for the MTs network in the different shapes for a sample size varying between 1 and 100. Right: difference of the mean for 1000 bootstrap repetitions of the anisotropy data for the MTs network with a sample size varying between 1 and 100. The original experimental data are presented in Fig. 2 of the main manuscript. (b) Left: p-values obtained for KS tests of 1000 bootstrap repetitions of the anisotropy data for and the actin network in the different shapes for a sample size varying between 1 and 100. Right: difference of the mean for 1000 bootstrap repetitions of the anisotropy data for the actin network with a sample size varying between 1 and 100. The original experimental data are presented in Fig. 3 of the main manuscript.

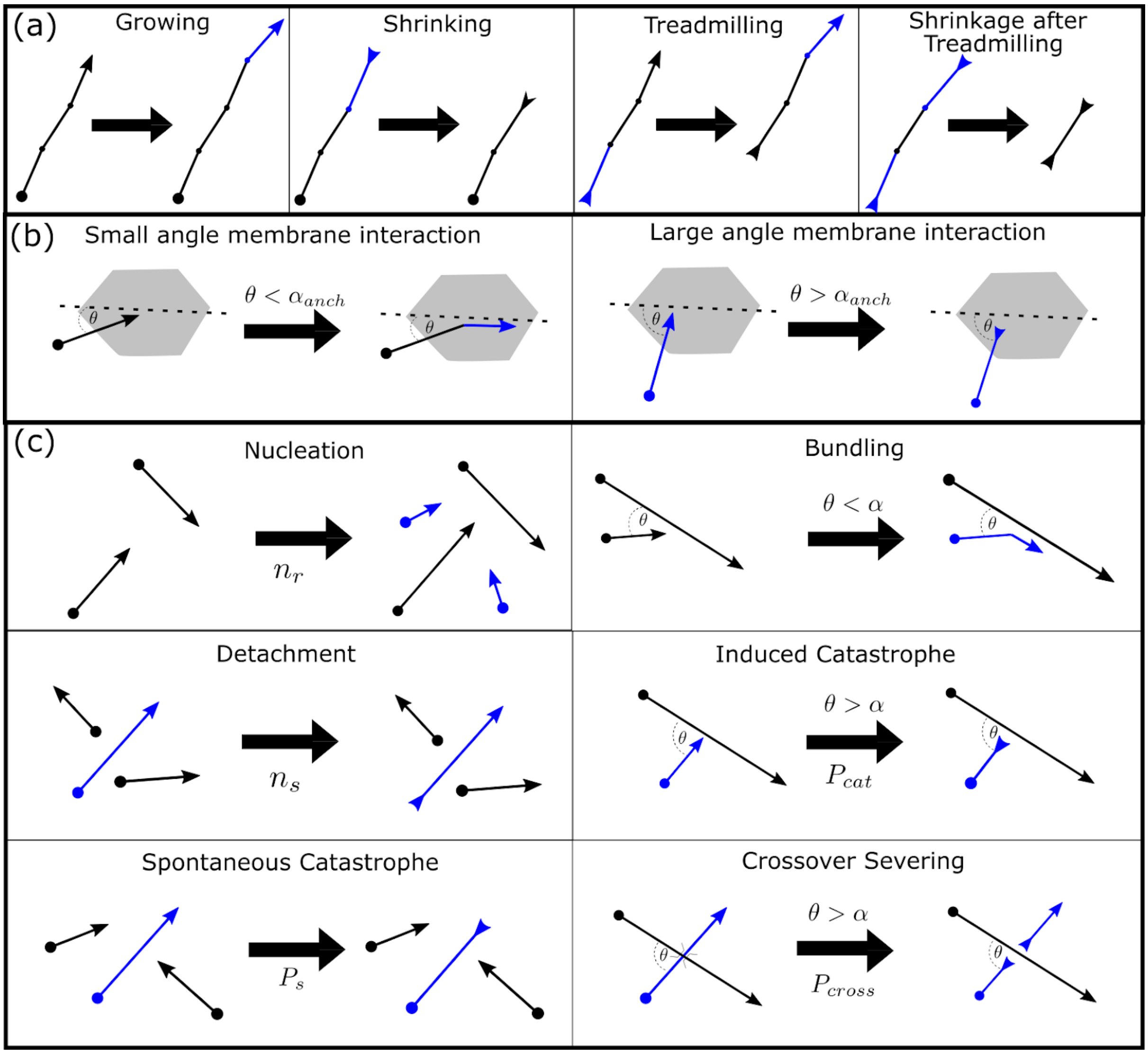

Figure S7: Diagram of different microtubule motions and interactions in the numerical simulations. Growing ends of microtubules are marked with outward arrows, inwards arrows mark shrinking ends and circles stationary ends (a) Microtubules represented as multi-segment vectors showing all possible states of the microtubule: growing, shrinking, treadmilling and shrinkage after treadmilling. For (b) and (c) whole microtubules are represented by distinct lines. (b) Microtubule interaction with the membrane. (c) Types of microtubule behaviour with the left column showing individual microtubule events and the right column showing microtubule-microtubule interactions.

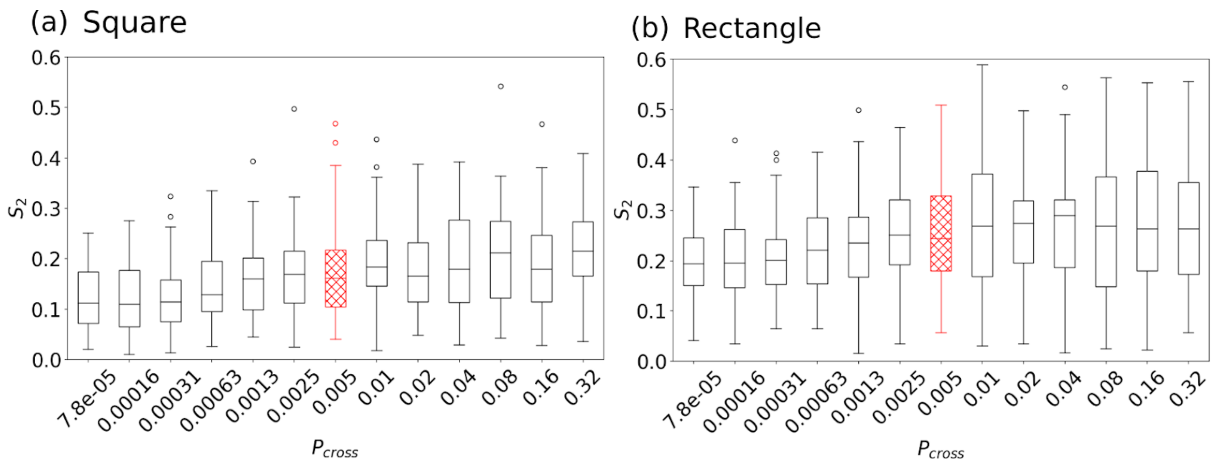

Figure S8: Computational simulations on (a) square domain and (b) rectangular domain; comparing anisotropy, S_2, as the probability of severing at crossovers (P_{cross}) is increased. The default with-severing [wild-type] simulation is marked in hashed red. Each simulation was run for t_{steps} =10000 time steps. Each bar represents R=50 simulations. A student t-test comparing each simulation to the wild-type simulation (red hashed) gives a p-value $\leq$0.05 when P_{cross} $\leq$0.00031 for both square and rectangle (and additionally in the square when P_{cross} =0.32).

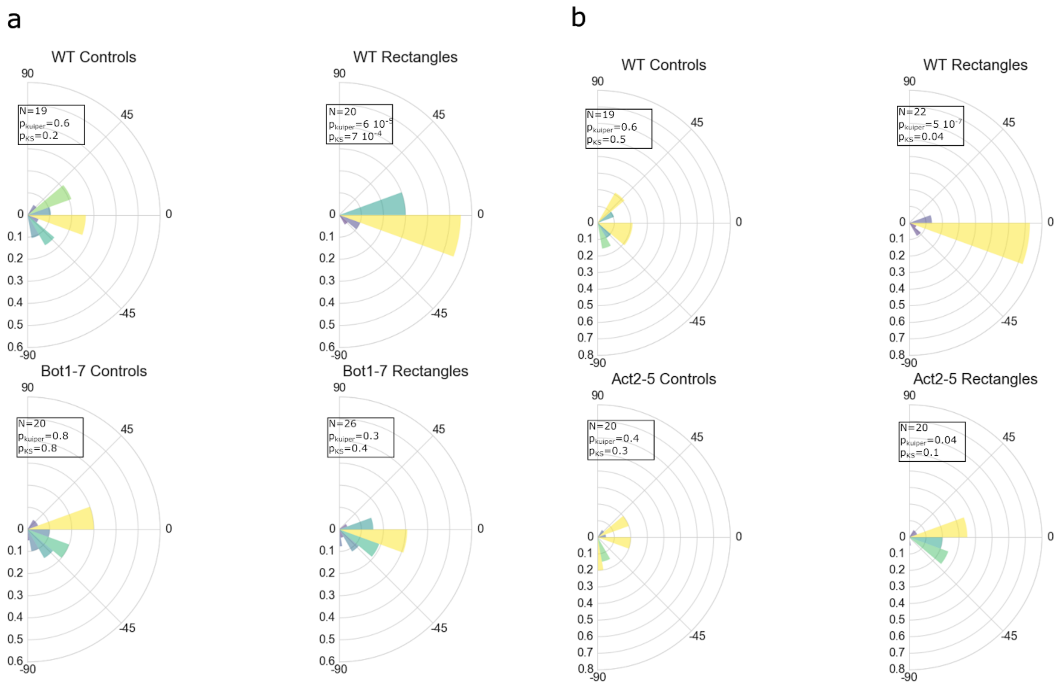

Figure S9: (a) Angular histograms of the average angle of the microtubule network in protoplasts in control geometry (left) and in rectangular microwells (right) for wild type protoplasts (top) and protoplasts in the *bot1-7* background mutant (bottom).
(b) Angular histograms of the average angle of the actin network in protoplasts in control geometry (left) and in rectangular microwells (right) for wild type protoplasts (top) and protoplasts in the *act2-5* background mutant (bottom).

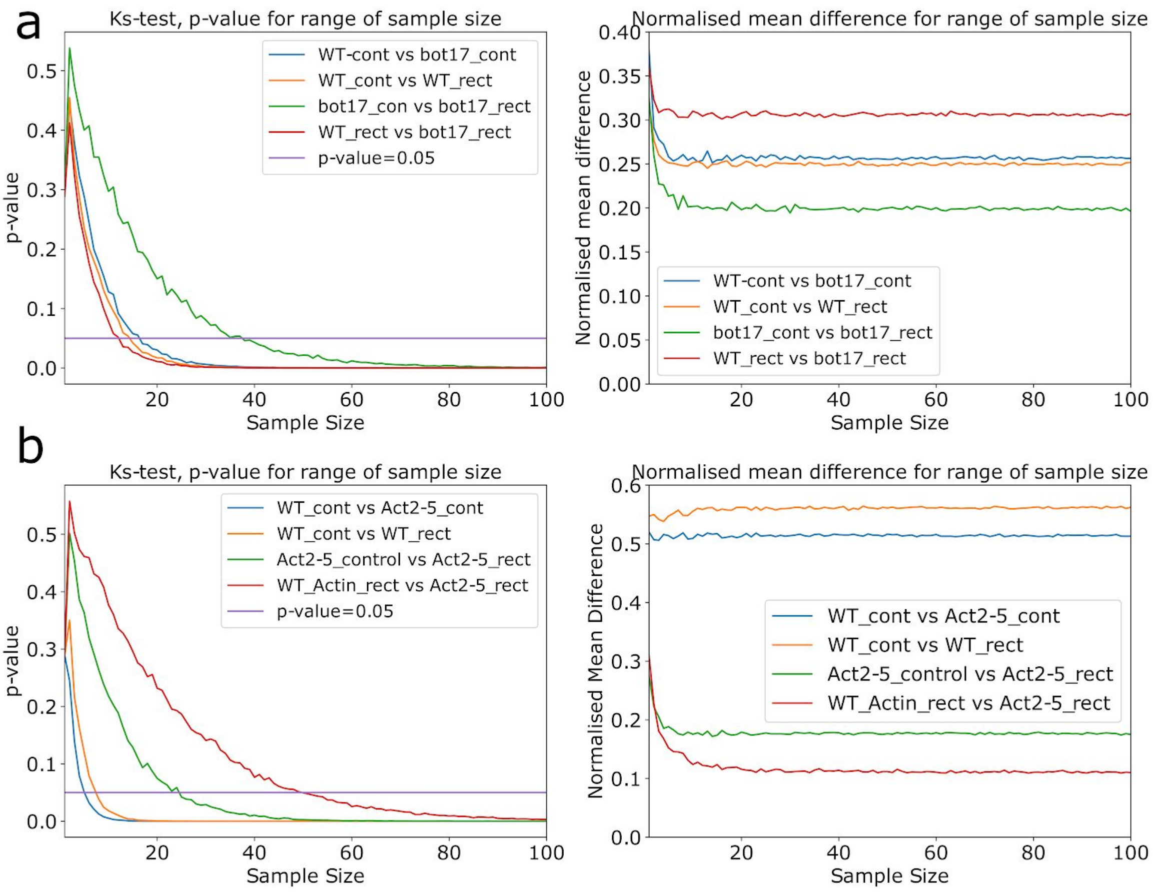

Figure S10: (a) Left: p-values obtained for KS tests between wild type protoplasts and *bot1-7* protoplasts of 1000 bootstrap repetitions of the anisotropy data for the MTs network in control and rectangular shapes for a sample size varying between 1 and 100. Right: difference of the mean for 1000 bootstrap repetitions of the anisotropy data for the MTs network with a sample size varying between 1 and 100. The original experimental data are presented in Fig. 4 of the main manuscript. (b) Left: p-values obtained for KS tests between wild type protoplasts and *act2-5* protoplasts of 1000 bootstrap repetitions of the anisotropy data for the actin network in control and rectangular shapes for a sample size varying between 1 and 100. Right: difference of the mean for 1000 bootstrap repetitions of the anisotropy data for the actin network with a sample size varying between 1 and 100. The original experimental data are presented in Fig. 5 of the main manuscript.

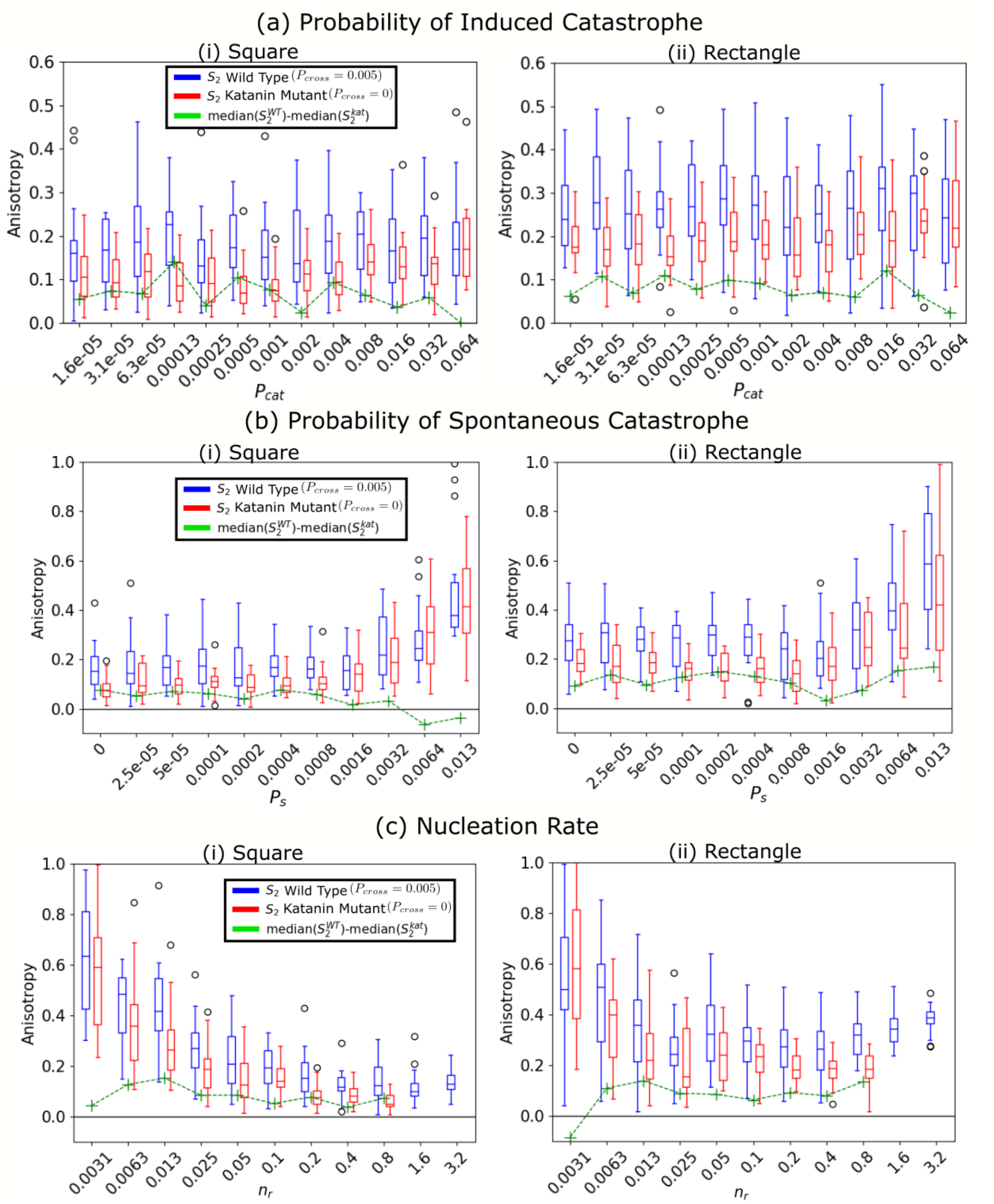

Figure S11: Computational simulations with t_{steps} =10000 on a (i) square domain and (ii) rectangular domain; comparing anisotropy, S_2, as (a) the probability of induced catastrophe P_{cat}, (b) probability of spontaneous catastrophe P_s and (c) nucleation rate n_r are varied. Each pair of bars represents the wild-type simulation of $P_{cross}=0.005$ on the left in blue and the katanin mutant simulation with $P_{cross}=0$ on the right in red (all other parameters are at their default value), with each bar representing R=20 simulations. The green line shows the difference in the median of the Wild-type (blue) and katanin (red) simulations. We use a student t-test to compare each pair of simulations. In (a) this gives a p-value$\leq$0.05 in (i) except a p-value=0.067 when $P_{cat}=1.6\times10^{-5}$ and at higher values of P_{cat} =0.016 and 0.064; (ii) except at the higher values of P_{cat} =0.002, 0.08, 0.032, 0.064. In (b) this gives a p-value$\leq$0.05 except when $P_s \geq 0.0016$ in both (i) and (ii). In (c) this gives a p-value$\leq$0.05 in (i) when $n_r=0.013, 0.05, 0.2, 0.4, 0.8$ and at the intermediate values of $n_r=0.1$ p-value=0.1 and $n_r=0.025$ p-value=0.051; (ii) at larger values of n_r when $n_r=0.05, 0.2, 0.4, 0.8$. No katanin (red) simulations are shown in (d) for large n_r as high nucleation rate combined with reduced severing leads to very high-density microtubules and thus long simulation times. No katanin (red) simulations are shown in (c) for large n_r as high nucleation rate combined with reduced severing leads to very high-density microtubules and thus long simulation times.

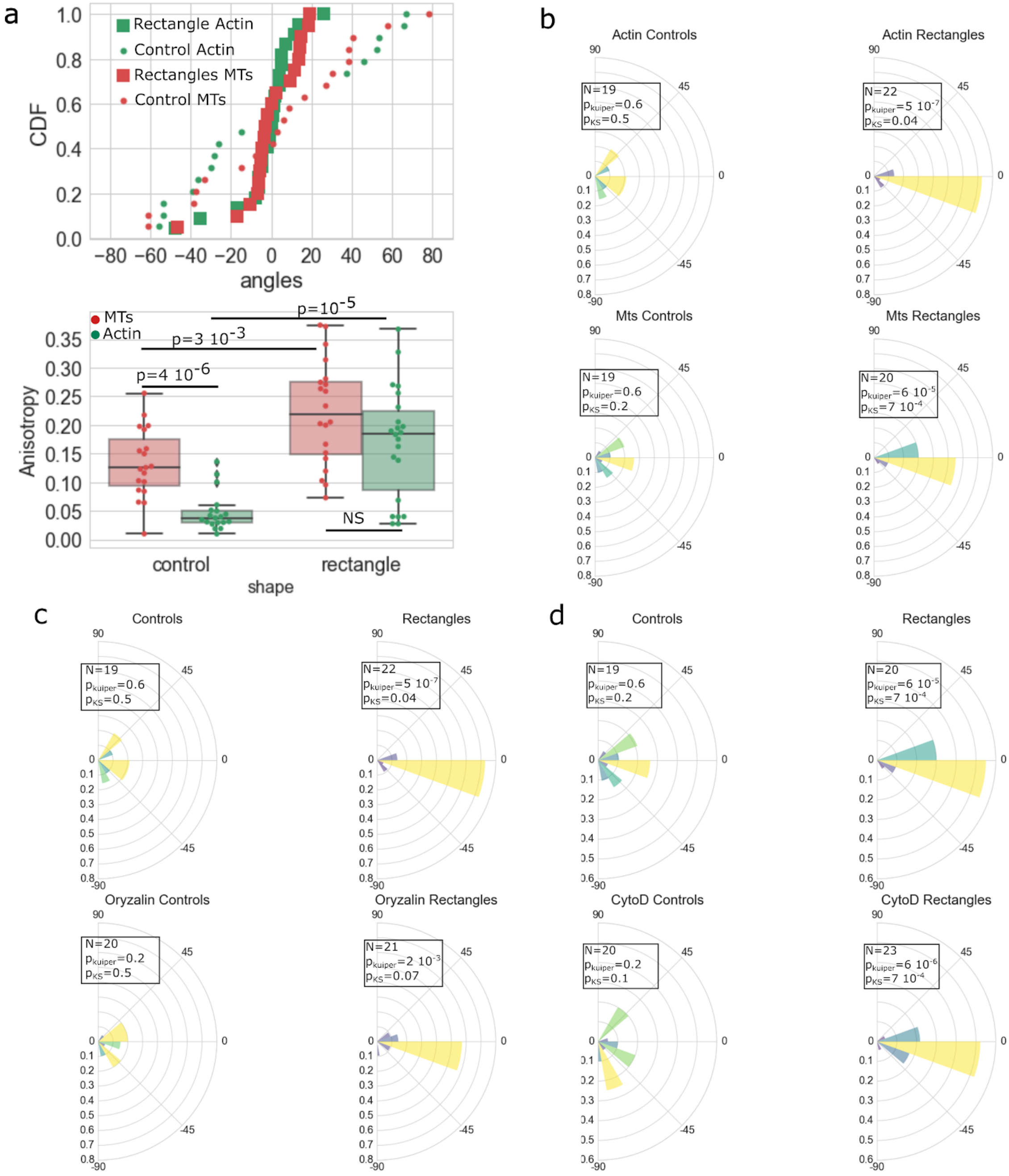

Figure S12: (a) Actin and microtubule networks are organised similarly in protoplasts confined in elongated geometry. Top: Cumulative distributions of the average angle of the actin (green) and microtubule (red) networks in rectangular microwells (square markers) and in control shape (spherical protoplasts. circle markers). Bottom: Box plot comparing the anisotropy of the microtubule and actin networks in rectangular and control shapes. The actin network is always less ordered than the microtubule network but in both cases, the networks are significantly more ordered in rectangular shapes than in spheres. (b) Angular histogram of the average angle of the actin (top) and microtubule (bottom) networks in protoplasts in control geometry (left) and in rectangular microwells (right). Both actin and microtubules organise along the main axis of the rectangular micro-wells. (c) Angular histograms of the average angle of the actin network in protoplasts in control geometry (left) and in rectangular microwells (right) for untreated protoplasts (top) and protoplasts treated with 20μM of the microtubule depolymerisation agent oryzalin (bottom). (d) Angular histograms of the average angle of the microtubule network in protoplasts in control geometry (left) and in rectangular microwells (right) for untreated protoplasts (top) and protoplasts treated with 4μM of the actin depolymerisation agent Cytochalasin D (bottom).

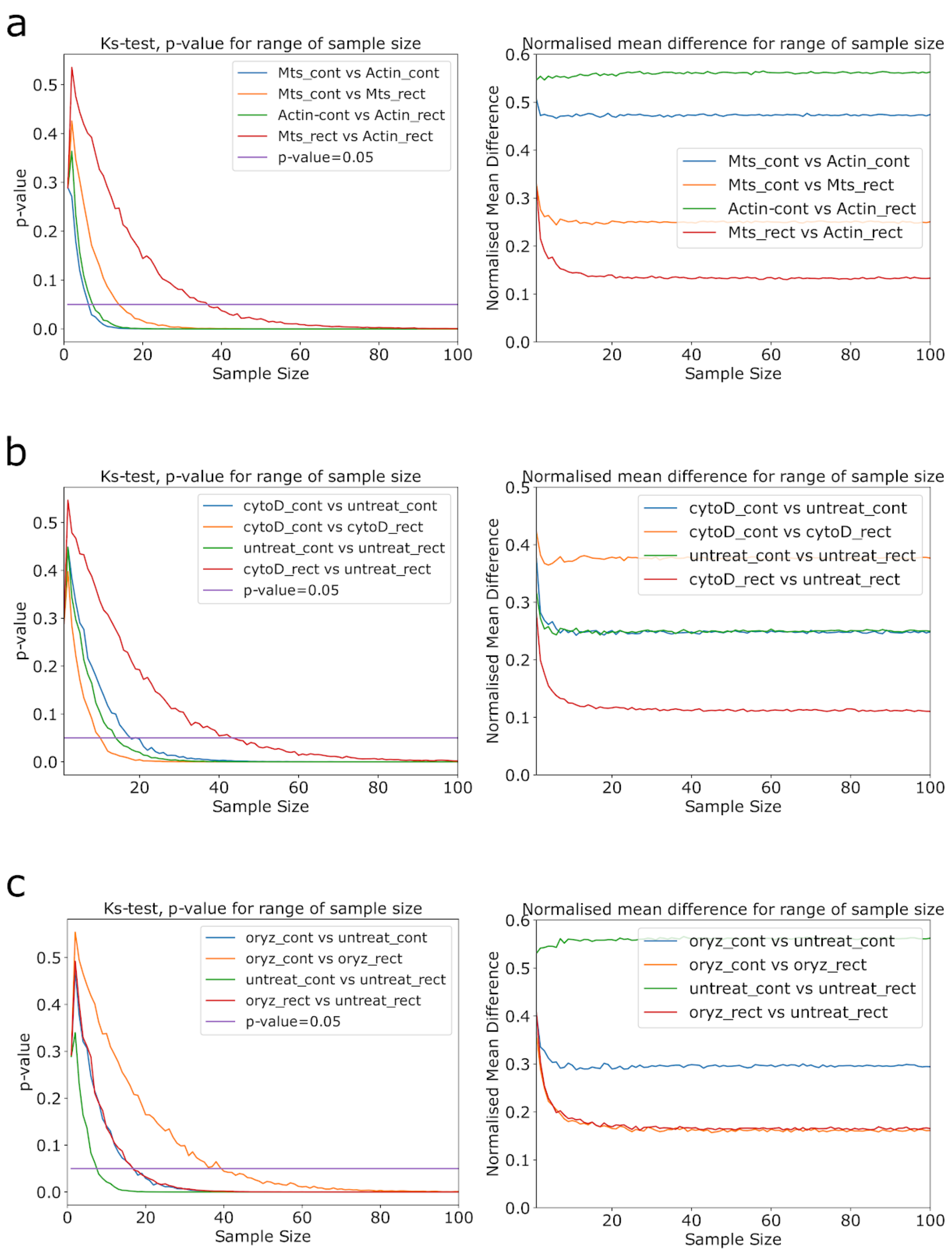

Figure S13:(a) Left: p-values obtained for KS tests between data for the anisotropy of the actin and MTs of 1000 bootstrap repetitions in control and rectangular shapes for a sample size varying between 1 and 100. Right: difference of the mean for 1000 bootstrap repetitions of the anisotropy data for the MTs and actin networks with a sample size varying between 1 and 100. The original experimental data are presented in Fig. S12. (b) Left: p-values obtained for KS tests between untreated protoplasts and protoplasts treated with CytochalasinD of 1000 bootstrap repetitions of the anisotropy data for the MTs network in control and rectangular shapes for a sample size varying between 1 and 100. Right: difference of the mean for 1000 bootstrap repetitions of the anisotropy data for the MTs network with a sample size varying between 1 and 100. The original experimental data are presented in Fig. 6 of the main manuscript. (c) Left: p-values obtained for KS tests between untreated protoplasts and protoplasts treated with Oryzalin of 1000 bootstrap repetitions of the anisotropy data for the actin network in control and rectangular shapes for a sample size varying between 1 and 100. Right: difference of the mean for 1000 bootstrap repetitions of the anisotropy data for the actin network with a sample size varying between 1 and 100. The original experimental data are presented in Fig. 6 of the main manuscript.

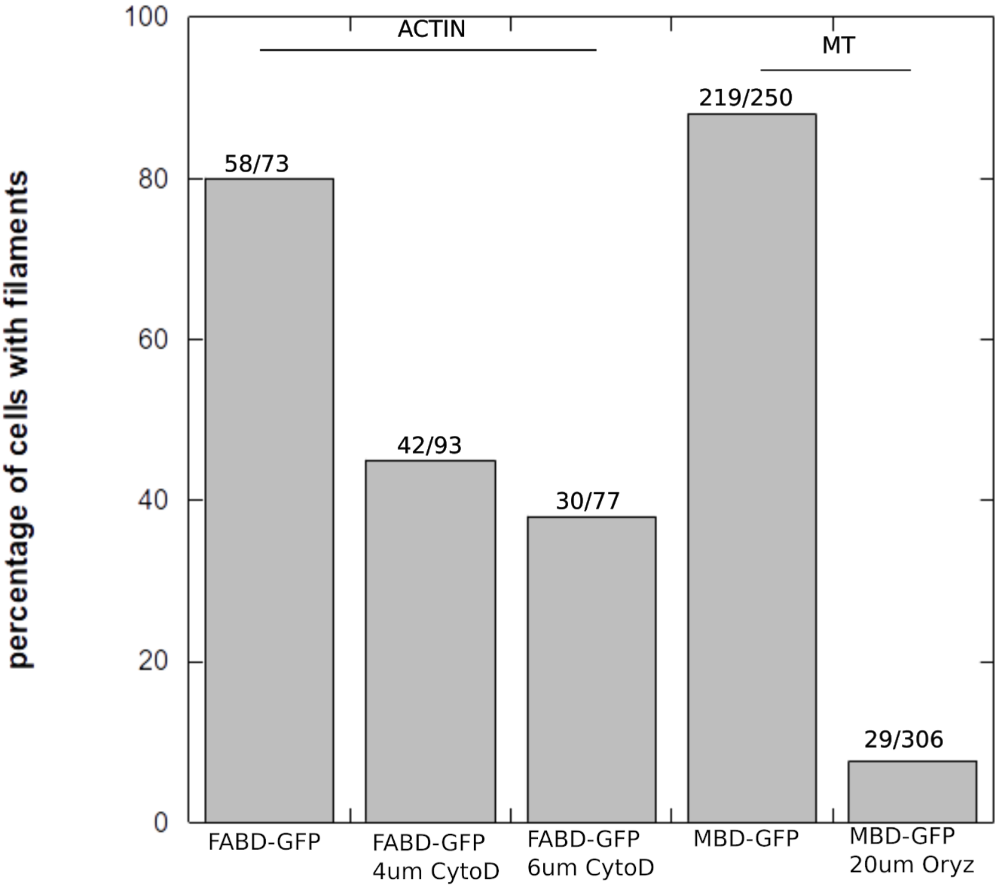

Figure S14: Number of protoplasts with apparent filaments after drug treatments. Protoplasts were generated, then treated with drugs for 30 min as described in the protocol in Materials and Methods, and then protoplasts were mounted on a hemocytometer for observation using a confocal microscope in order to check the presence of actin filaments (in FABD-GFP lines) or microtubules (in MBD-GFP lines).

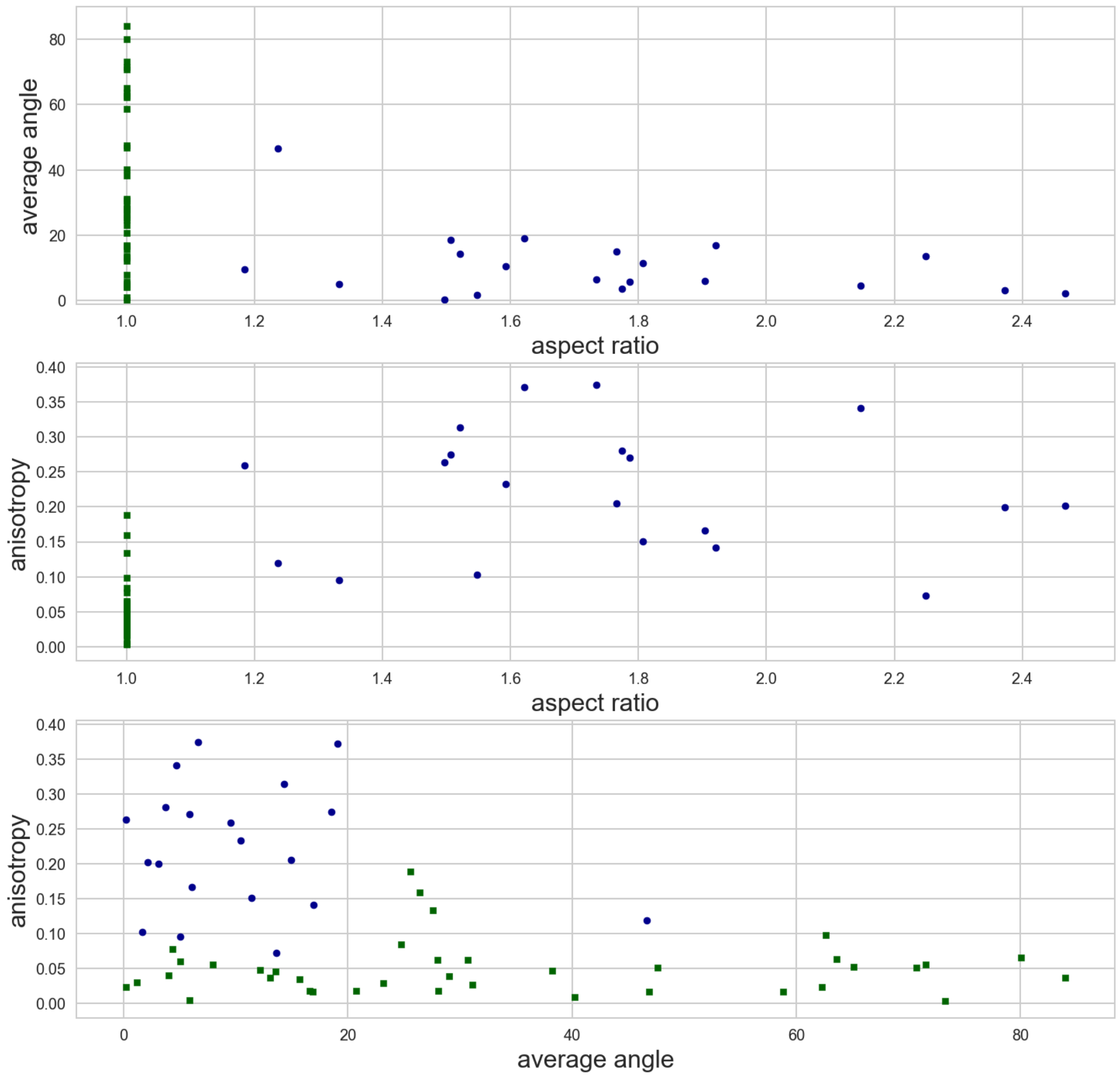

Figure S15:
Evolution of the average angle (upper graph) and anisotropy (middle graph) of the MT network as a function of the aspect ratio of the protoplasts (aspect ratio of 1 corresponds to protoplasts confined in square shapes). Lower graph: anisotropy and average angle of the MT network in protoplasts confined in rectangular shape (circle markers) and square shape (square markers).

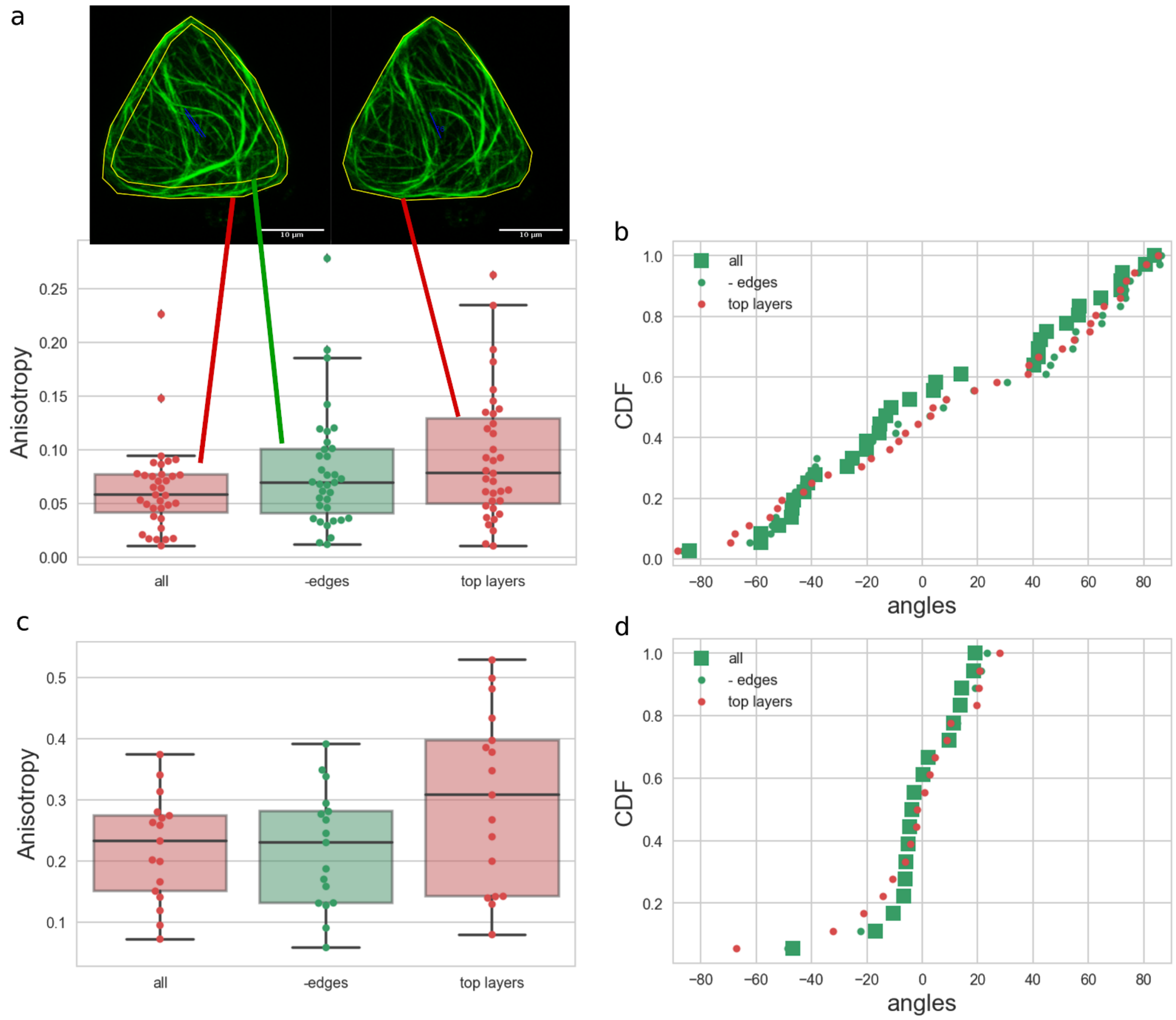

Figure S16: Comparison of the quantification of the microtubule network organisation in protoplasts in triangular shapes (a and b) and rectangular shapes (c and d).

(a and c) Anisotropy distributions obtained from Fibriltool analysis on whole pictures (all focus plans from z-stack projected onto one plane), pictures with the border of the cells removed (to see the effect of accumulation at the border of the shape due to the maximum intensity projection) and when only the 10 top focus planes from the z-stacks were taken for the maximum intensity projection.

(b and d) Distribution of the average angles obtained from Fibriltool analysis on whole pictures, pictures without borders, or pictures with top layers only.

For all cases, the distributions between the 3 types of analysis (all, -edges or top layers) did not present significant differences when statistical tests were performed.

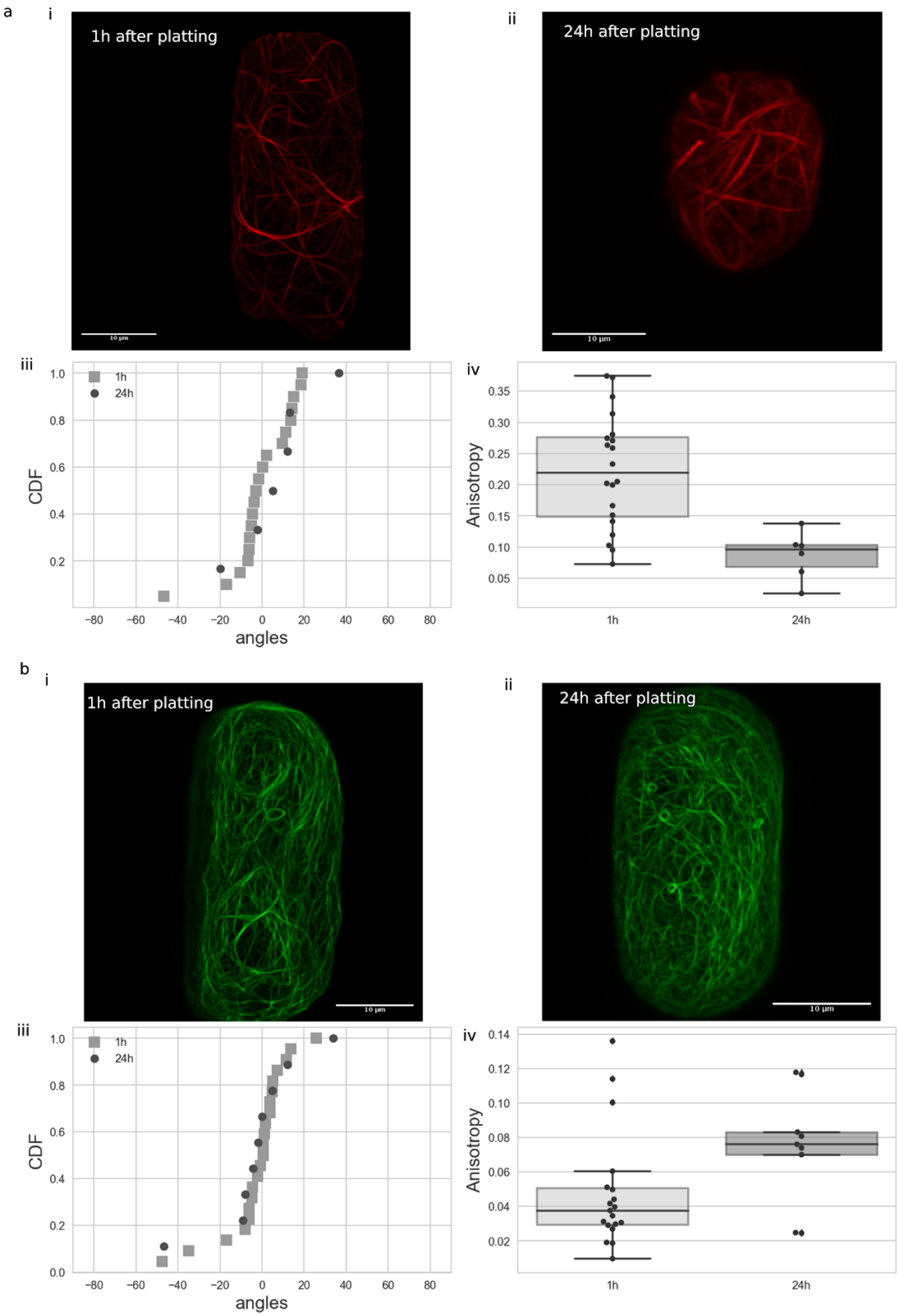

Figure S17:
Quantification of the organisation of the microtubule (a) network and actin (b) network in protoplasts confined in rectangular shapes at 1h or 24h after platting.

Supplementary Table1:

**Parameter**	**Symbol**	**Default Value**	**Reference**
Bundling angle threshold	α	0.7 radians	(2)
Weak anchoring angle	α_{anch}	0.7 radians	(3)
Probability of catastrophe	P_{cat}	0.001	Effect presented in Fig. S11 and discussed in SI methods
Probability of cutting crossing microtubule	P_{cross}	0.005	(4) Effect presented in Fig. S8 and discussed in methods
Length of computational temporal step	t_{sim}	$\approx 0.1 - 0.2s$	*(3)*
Number of time steps	t_{steps}	3000 or 10000	(3)
Number of repeats	R	50 or 20	
Random microtubule shrinkage from plus-end	P_s	0	(3) Effect presented in Fig. S11 and discussed in SI methods
Initial nucleations	n_{init}	30	
Probability of detachment	n_s	0.001 per nucleation site per time step	(3)
Nucleation rate	n_r	0.2 nucleations per time step	(2) Effect presented in Fig. S11 and discussed in SI methods
Nucleation rate per time step per unit surface are	n_p	$\approx$1 nucleation per time step per surface area	(2) discussed in SI methods
Interaction distance	d	49nm	(3)

Table Supplementary 1: Computational parameters used in the numerical simulations

Supplementary Material and Methods:

Plant crosses

10-day old seedlings were transferred into soil and plants were grown under continuous light at 20ºC until flowers appeared, before crossing. For the purpose of this study, *act2-5* plants were crossed with FABD-GFP plants and *bot1-7* plants were crossed with MBD-GFP plants (generated plants were homozygous for the mutant only).

Protoplast generation

To generate callus cultures, roots from 2 weeks old seedlings were collected, chopped into thin sections, and then transferred onto solid callus induction medium (3.8g/L B5 salt mix, 25g/L glucose, 0.625 g/L MES, 1.25mL Gamborg vitamins, pH adjusted to 5.7 with KOH, 62.5 µg/L kinetin, 625 µg/L 2,4D, 10g/L Phytoagar) at 25 °C. The calli were then transferred to a new medium every 2 weeks.

Protoplasts were obtained as previously described (5) by a combination of cell wall degradation and hypo-osmotic shock. Packed cells were gently mixed, in a 15 ml tube, with 5.5 mL of enzyme solution containing 2 mM CaCl2, 2mM MgCl2, 10mM MES, 1 mM L-ascorbic acid, pH 5.5 with KOH, 17 mg/mL Cellulysin (Calbiochem, La Jolla, CA), 17 mg/mL Cellulase RS (Yakult, Co. Ltd., Tokyo, Japan), 0.4 mg/mL Pectolyase Y-23 (Seishin Pharmaceutical Co. Ltd., Nihombashi, Japan), 3.5 mg/mL Bovine Serum Albumin (Sigma, St. Louis, MO), and 600 mOsm with mannitol, sterilised by filtration. Cells were then incubated for 2 hours with rotation shaking (60 rpm) at 25ºC. After 3 min of centrifugation at 800 rpm, the supernatant was discarded and the cells were resuspended in 5 mL of washing medium for 5 min (2 mM CaCl2, 2 mM MgCl2, 10 mM MES, pH 5.5 with KOH, 600 mOsm with mannitol). Cells were pelleted again (3 min 800 rpm), the supernatant was removed and 5 mL of hypoosmotic medium (same as washing medium, osmolarity 280 mOsm with mannitol) was added to release protoplasts. After 15 min of gentle shaking (30 rpm), protoplasts were sorted from aggregates by filtration through a 75 µm mesh. Fresh protoplasts were transferred into culture medium PCM consisting of: 0.5MS supplemented with IAA 2.0 mg/l 2,4-D 0.5mg/l IPAR 0.5 mg/l and 0.4 M glucose (6) and then directly loaded on the microwell array.

We expect that the protoplasts did not regenerate their walls in the time of the experiments (which never exceeded 6 hours after plating except when otherwise specified), as the synthesis of a new cell wall generally takes 24 h after cell wall removal (7, 8) or 3 days in our system (Fig. S3).

Microwell fabrication

Microchambers were fabricated following standard microfabrication techniques (9). First, shapes of interest were designed with AutoCAD. The CAD file used for this study is available in: https://gitlab.com/slcu/teamHJ/publications/durand_etal_2019

The protoplasts generated with the protocol described here presented diameters of about 25±8 µm on average (standard error). For each shape, we designed moulds with diameters between 15 and 40 µm. A silicon master with silicon features was created in a cleanroom following standard micro-lithography techniques. The features were fabricated with a height of 20 µm±0.5µm (Fig. S2). The height of the features was measured with a scanning electron microscope (FEI Sirion). The silicon wafers were then rinsed with ethanol and water

and air-dried. PCM medium mixed with 1.5% agarose was then poured over the silicon wafer and let cool to gel. Once gelled, the agarose microwells were carefully detached from the wafer.

The microwell array was then deposited on PDMS posts located at the bottom of a plastic petri dish. Fresh PCM was poured such that the air/liquid interface almost reaches the top of the microwells. This was to prevent the agarose from drying throughout the experiment. The protoplasts were then plated on top of the microwells, some protoplasts migrated to the bottom of a microwell by sedimentation (about 1 hour of sedimentation). Protoplasts exhibited shapes dictated by the wells and showed polymerised cytoskeletons several hours following plating, indicating that their general physiology was not perturbed. Only protoplasts that were deformed inside the micro-chambers were taken into account for analysis. Indeed, small protoplasts that fit at the bottom of the chambers without being deformed into square, triangle or elongated shape, remained spherical.

Once the protoplasts were plated into the micro-chambers, a coverslip was carefully added on top of the array before imaging under an upright microscope (Fig. 1). The protoplasts are usually observed 30 min to 1h after being plated, thus the organisation we observed forms on a time scale faster than hours. Moreover, the specific organisation observed here in case of the rectangular shape is persistent in time and could still be observed 24h after the protoplasts were plated (Fig. S17). In our experiments, we thus monitor the steady-state organisation of the cytoskeletal network.

Confocal image acquisition and image analysis

Protoplasts lodged in micro-chambers were imaged with a Zeiss 780 or Zeiss 880 Airyscan confocal laser scanning microscope with a 63X oil objective and Z-stacks of cells with 0.18 µm intervals were obtained for 3D reconstruction. Images taken with the 780 confocal were processed with ImageJ (10), http://reb.info.nih.gov/ij).

Zen 2.3 software was used to process the images acquired with the 880 Airyscan confocal microscope. The software processes all Airy channels in order to obtain images with enhanced spatial resolution in 3D (11).

After processing, z-stacks were projected on one plane with maximum intensity projection with ImageJ to create 2D images. 3D analysis of the networks was not possible in the conditions of our experiments since the signal in the deeper layers of the cells was not sufficient (SI Fig. S2). The FIJI plugin Fibriltool (12) was used in order to quantify the average orientation and the anisotropy of the network. The average orientation gives information on the direction of the network, while the anisotropy gives an estimation on how well the filaments are aligned with each other.

Regions of interest were drawn manually to define the contours of the cells on the 2D pictures and nematic tensors of MT and actin filament arrays inside the region of interest (ROI) were obtained using FibrilTool. Fibriltool directly provides the average orientation of the network inside the ROI and the anisotropy of the network with a score between 0 and 1 for every cell.

The anisotropy is given by $q = n_1 - n_2$ where n_1 and n_2 are the eigenvalues of the nematic tensor **n** with $\mathbf{n} = \mathbf{t} \otimes \mathbf{t}$. The unit vector $\mathbf{t} = (t_x, t_y) = \frac{(\partial I/\partial y, -\partial I/\partial x)}{\sqrt{(\partial I/\partial x)^2 + (\partial I/\partial y)^2}}$ is the tangent to the putative fibrillar structures and $I(x,y)$ is the pixel intensity level in the picture.

Further information on the validation of the quantification extracted from the pictures is provided (SI Fig. S16).

Statistical analysis

The KS tests (and t-tests) used are the ones currently in the Python Scipy library (13). In addition, we used a bootstrap method where each distribution was reproduced 1000 times by drawing randomly with replacement from the original data set (the sample size of the random draw was varied between 1 and 100 in order to estimate the effect of the sample size). To take into account the circular lattice nature of the distribution of the average angles, Kuiper tests were run on the angles data set. To that end, uniform distributions of the same number of angles as in the compared dataset were generated and a two-sample Kuiper test was run (code available at https://github.com/aarchiba/kuiper/blob/master/kuiper.py). Results of KS tests on the same data sets are provided for information in SI figures.

Numerical simulation description and parameter choice

The numerical model is an adaptation of that developed in (3) (see also Fig. S7). Each tubulin section contained within a MT is modelled as a unit length vector, with MTs as multi-segment vectors. The plus-end of a MT is marked to grow or shrink and the minus-end to shrink or remain static. Shrinking removes successive unit vectors from the corresponding MT end. Growth adds a unit vector to the corresponding end, with the direction matching that of the previous tubulin modified by a small proportion of randomness. MTs are contained within a defined domain and prevented from crossing the membrane either by redirecting their growth direction along the membrane (before the addition of the random component) if the angle between the surface and the MT is less than α_{anch}; or by a catastrophe such that the MT starts shrinking if the angle between the surface and MT is bigger than α_{anch}. MTs can re-enter the cell interior after contact with the membrane due to the random element of the growth direction.

MTs were nucleated 80nm within and parallel to the cell surface, at a constant rate, n_p, per time step per unit surface, where as default we take $n_p \approx 1$ (corresponding to $n_r = 0.2$ and $\approx 0.005\ \mu m^{-2} s^{-1}$). This is a similar order of magnitude to other computational studies of 0.001 s^{-1} μm^{-2} in (2) and 0.0002 s^{-1} μm^{-2} in (14)) and Fig. S11-c further examines different nucleation rates. The MT minus-end detaches with probability, n_s, per nucleation site per time step, at which stage the MT starts shrinking from the minus-end causing treadmilling or shrinkage depending on plus-end behaviour. The MT can spontaneously catastrophe with probability P_s, where we assume $P_s = 0$ for simplicity. Previous computational work has taken the probability of spontaneous catastrophe at 0.003 and (default) 0.0045 s^{-1} per microtubule (2, 14), which corresponds to $P_s \approx 0.0003 - 0.0009$, and Fig S11b examines different rates of induced catastrophe further.

For MT interactions Mirabet et al (3) imposed that when a growing MT is within distance d of the next closest MT if the angle between them is less than α it bundles with the direction of the new unit vector tubulin aligning with the neighbouring MT, but if the angle is greater than α an induced catastrophe occurs and the growing end changes to shrinking. Rather than always causing an induced catastrophe when the MTs interact at angles greater than α, we introduced a probability $(1 - P_{cat})$ that the MTs continues to grow but will be cut at the crossover at a later time with probability P_{cross} per tubulin per time step. In more detail,

when a MT plus end undergoes a growth event, we first determine if its nearest neighbour MT is within distance d (measuring from MT centre lines). If it is, and the angle is below the threshold α the MT bundles with its growth direction changing to match that of the existing MT (following (3)). If the angle is larger than the threshold, we assume this MT will be crossing the neighbouring MT. This causes an induced catastrophe with probability P_{cat} , in which case the MT growing plus-end changes to shrinking. Otherwise, the MT keeps growing and this element is added to a cut list, and the nearest-neighbour tubulin element that we assume is the one crossed is also recorded. At each growth step, every element in the cut list is severed with a probability P_{cross} but only if its nearest-neighbour tubulin element still exists (this ensures the MT will not be severed if the MT it crossed over has been destroyed). Severing breaks the MT into two at the cut element, with both MTs shrinking from their cut ends (Fig. S7). Given that the model is fully 3D and MT adjacency is defined by a distance threshold, this method allows adjacent unit vectors on the same MT to be recorded as crossing for the same cross-over (so tested for induced catastrophes or added to the cut list). Thus, relatively small probabilities can correspond to relatively large levels of cutting or induced catastrophes, for example, if $P_{cat} = 0.001$ then assuming a MT is checked for an induced catastrophe at 10 unit vectors during a cross-over then the probability of an induced catastrophe at that MT crossover is $1-(1-0.001)^{10} \approx 0.01$, nearly ten times larger. We also tested the alternative to computationally impose that only the first tubulin in a continuous line of tubulin classed as a crossover is marked for an induced catastrophe or for cutting, leading to a decrease in the number of elements in the cut-list and a shallower anisotropy trend with increasing cut probability, but no change to the conclusion within this paper. This condition is not imposed for any of the simulations presented in this paper. As a default, we take $P_{cat} = 0.001,$ with Fig. S11-a showing a winder parameter sweep of this variable.

For our with-severing simulations representing the wild-type we use $P_{cross} = 0.005$ and for the without-severing simulation representing the katanin mutant, we take $P_{cross} = 0$ (and keeping all other parameters consistent). For our wild-type, this corresponds to an average time until severing after a crossover of $t_{sim}/P_{cross} \approx 20-40s$ (slightly overestimated due to recording the same crossover at multiple positions) which is of similar magnitude to that observed experimentally by (4) in *Arabidopsis* of $41s \pm 14s$ in hypocotyl cells and $109 \pm 80s$ in pavement cells. Our katanin mutant choice of $P_{cross} = 0$ is consistent with (4), where, in their katanin mutant no severing was observed at any of the 1030 crossovers.

Our simulations were run for $t_{steps} = 10000$ or $t_{steps} = 3000$ steps. With a simulation time step, $t_{sim} \approx 0.1-0.2,$ this corresponds to a real time of approximately 15-30min and 5-10 minutes respectively. The number of repeats per parameter set was $R = 50$ or $R = 20$.

To calculate MT anisotropy level and direction we analyse the simulation output of microtubule positions in Python. To compare to anisotropy calculations on 2D experimental images, we compute MT anisotropy in 2D, on two opposite faces of our domain. Tubulin directional unit vectors are projected onto the plane of the face to reduce them to 2D. The anisotropy measure is the S_2 order parameter (15)

$$S_2 = \frac{\sqrt{\left[\sum_m cos(2\theta_m)\right]^2 + \left[\sum_m sin(2\theta_m)\right]^2}}{M},$$

where M is the total number of tubulin unit vectors over all the MTs and θ_m the angle between the unit vector and a reference direction, where $0 \le S_2 \le 1$ and $S_2 = 0$ corresponds to isotropy and $S_2 = 1$ to perfect alignment. The orientation of alignment (15) is calculated as the angle

$$\Theta_{S_2} = tan^{-1}\left(\frac{\sum_m sin(2\theta_m)}{\sum_m cos(2\theta_m) + MS_2}\right),$$

where $-\pi/2 < \Theta_{S_2} \le \pi/2$ (with 0 corresponding to alignment with the reference direction and $\pi/2$ at right angles to it).

Supplementary References:

1. R. Rezakhaniha, *et al.*, Experimental investigation of collagen waviness and orientation in the arterial adventitia using confocal laser scanning microscopy. *Biomech. Model. Mechanobiol.* **11**, 461–473 (2012).
2. S.H. Tindemans, E.E. Deinum, J.J. Lindeboom, B.M. Mulder, Efficient event-driven simulations shed new light on microtubule organization in the plant cortical array. *Front. Phys.* **2**, (2014) doi:10.3389/fphy.2014.00019.
3. V. Mirabet, *et al.*, The self-organization of plant microtubules inside the cell volume yields their cortical localization, stable alignment, and sensitivity to external cues. *PLoS Comput. Biol.* **14**, e1006011 (2018).
4. Q. Zhang, E. Fishel, T. Bertroche, R. Dixit, Microtubule severing at crossover sites by katanin generates ordered cortical microtubule arrays in Arabidopsis. *Curr. Biol.* **23**, 2191–2195 (2013).
5. P. Durand-Smet, *et al.*, A comparative mechanical analysis of plant and animal cells reveals convergence across kingdoms. *Biophys. J.* **107**, 2237–2244 (2014).
6. J. Mathur, C. Koncz, L. Szabados, A simple method for isolation, fiquid culture, transformation and and regeneration of Arabidopsis thaliana protoplasts. *Plant cell reports* 221–226 (1995).
7. T. Nagata, I. Takebe, Cell wall regeneration and cell division in isolated tobacco mesophyll protoplasts. *Planta* **92**, 301–308 (1970).
8. A.E. Cuddihy, P.J. Bottino, Winged-bean protoplasts: isolation and culture to callus. *Plant Cell Tissue Organ Culture* **209**, 201–209 (1982).
9. D.B. Weibel, W.R. Diluzio, G.M. Whitesides, Microfabrication meets microbiology. *Nat. Rev. Microbiol.* **5**, 209–218 (2007).
10. J. Schindelin, *et al.*, Fiji: an open-source platform for biological-image analysis. *Nat. Methods* **9**, 676–682 (2012).
11. K. Korobchevskaya, B. Lagerholm, H. Colin-York, M. Fritzsche, Exploring the potential of airyscan microscopy for live cell imaging. *Photonics* **4**, 41 (2017).

12. A. Boudaoud, *et al.*, FibrilTool, an ImageJ plug-in to quantify fibrillar structures in raw microscopy images. *Nat. Protoc.* **9**, 457–463 (2014).

13. E. Jones, T. Oliphant, P. Peterson, SciPy: Open source scientific tools for Python. (2001).

14. J.J. Lindeboom, *et al.*, Cortical microtubule arrays are initiated from a nonrandom prepattern driven by atypical microtubule initiation. *Plant Physiol.* **161**, 1189–1201 (2013).

15. E.E. Deinum, S.H. Tindemans, J.J. Lindeboom, B.M. Mulder, How selective severing by katanin promotes order in the plant cortical microtubule array. *Proc Natl Acad Sci USA* **114**, 6942–6947 (2017).
